# Cross-talk among novel programmed cell death pathways: a decisive network in renal ischemia-reperfusion injury

**DOI:** 10.3389/fimmu.2026.1844850

**Published:** 2026-06-18

**Authors:** Yalin Lou, Jueheng Wu, Shilei Cheng, Guanghan Wu, Liang Guo, Fan Yang, Jianbo Wu

**Affiliations:** 1 Department of Anesthesiology, the First Affiliated Hospital of Shandong First Medical University & Shandong Provincial Qianfoshan Hospital, Shandong Institute of Anesthesia and Respiratory Critical Medicine, Shandong Provincial Clinical Research Center for Anesthesiology, Jinan, China; 2 School of Anesthesiology, Shandong Second Medical University, Weifang, China; 3 Department of Anesthesiology, The Second Affiliated Hospital of Dalian Medical University, Dalian, China; 4 Department of Anesthesiology, Liaocheng People’s Hospital, Liaocheng, China; 5 Cheeloo College of Medicine, Shandong University, Jinan, China

**Keywords:** acute kidney injury, crosstalk, programmed cell death, renal ischemia-reperfusion injury, targeted therapy

## Abstract

Renal ischemia-reperfusion injury (IRI) is a major pathological driver of acute kidney injury (AKI), for which effective therapeutic strategies remain lacking. Recently, novel forms of programmed cell death (PCD), including pyroptosis, ferroptosis, cuproptosis and necroptosis, have been identified as key mediators of tubular damage and inflammation in renal IRI. This review delineates a hierarchically organized and spatiotemporally regulated PCD network, positioning it as a central determinant of cellular fate following renal IRI. We systematically characterize the distinct molecular signatures of each death modality and critically examine their extensive crosstalk within the pathological renal microenvironment. Synthesizing current evidence, we demonstrate that this network operates in a cell type- and phase-specific manner, driving a vicious cycle of inflammation and oxidative stress. Targeting this interconnected network rather than isolated pathways represents a paradigm shift. We critically assess current therapeutic strategies and their limitations in the context of this network. Finally, we propose a forward-looking roadmap that emphasizes combination therapies guided by spatial transcriptomics, patient stratification using PCD-specific biomarkers and the development of smart nanosystems capable of dynamically modulating key network nodes. Deciphering and therapeutically intervening in this PCD network is pivotal for developing effective treatments for renal IRI.

## 1 Introduction

Renal ischemia-reperfusion injury (IRI) is a pathological phenomenon in which tissue damage is exacerbated upon restoration of blood flow following an ischemic event. In severe cases, it may lead to irreversible renal injury (1, 2). Clinically, renal IRI is primarily manifested by elevated serum creatinine (Scr) and blood urea nitrogen (BUN), oliguria or anuria, and may be accompanied by complications such as edema, hypertension, hyperkalemia, metabolic acidosis and infections. In severe instances, it can progress to acute renal failure (3). The kidney receives an exceptionally rich blood supply, accounting for approximately 20-25% of cardiac output. This high-perfusion characteristic renders it particularly vulnerable to ischemia. In recent years, with the widespread application of technologies such as cardiopulmonary bypass, arterial bypass grafting and organ transplantation, the incidence and mortality of acute kidney injury (AKI) induced by renal IRI have been rising annually (4). Renal IRI poses a significant clinical challenge and underlies the development of AKI in various contexts ranging from cardiac surgery to renal transplantation (5). Paradoxically, the restoration of blood flow triggers oxidative stress and inflammatory cascades. These events ultimately lead to the death of renal tubular epithelial cells and consequent organ dysfunction. Owing to the complexity and redundancy of the injury pathways, therapeutic translation has remained unsatisfactory despite decades of research efforts. Previous studies have predominantly focused on apoptosis and autophagy. Although these processes are crucial, clinical translation based on their regulation has been limited. This suggests the involvement of other key cell death mechanisms in the complex pathology of renal IRI.

Based on their regulatory mechanisms, cell death can be classified into two major categories, namely accidental cell death and regulated cell death (6). Accidental cell death, also known as cell necrosis, is a non-programmed, unregulated form of death directly caused by intense physical, chemical or biological damage. This process leads to cellular rupture and the release of intracellular contents, typically accompanied by a significant inflammatory response. In contrast, regulated cell death is precisely controlled by specific intracellular signaling pathways and can be modulated through genetic or pharmacological interventions. The past decade has witnessed the discovery and characterization of several novel, genetically regulated forms of programmed cell death (PCD), including pyroptosis, ferroptosis, cuproptosis and necroptosis (7–9). Each is defined by a distinct molecular mechanism. Pyroptosis occurs via Gasdermin pore formation activated by inflammasomes. Ferroptosis proceeds through iron-dependent lipid peroxidation. Cuproptosis is driven by copper-induced mitochondrial proteotoxic stress. And necroptosis is executed by the RIPK1/RIPK3/MLKL axis. Here, RIPK1 stands for receptor-interacting serine/threonine-protein kinase 1, RIPK3 for receptor-interacting serine/threonine-protein kinase 3 and MLKL for mixed lineage kinase domain-like protein. Compelling evidence now places these pathways at the center of the pathogenesis of renal IRI (10).

However, viewing these death programs as isolated, parallel pathways is an oversimplification that may constrain therapeutic efficacy. Within the intricate microenvironment of the post-ischemic kidney, these pathways engage in intensive molecular crosstalk. They thereby form an integrated cell death network (11). This network exhibits properties of redundancy, priming and switch-like behavior. Inhibiting one pathway may divert cellular fate to another, while activating one can explosively ignite others. Therefore, comprehending the network’s architecture and logic is paramount. This includes understanding its key hubs, positive feedback loops and cell-type-specific connections.

A comprehensive literature search was performed in the PubMed and Google Scholar databases for articles published up to the present. The search terms included combinations of several keywords. These included “renal ischemia-reperfusion injury”, “acute kidney injury”, “programmed cell death”, “pyroptosis”, “ferroptosis”, “cuproptosis”, “necroptosis”, “autophagy”, “apoptotic”, “PCD network”, “therapeutic targeting”, “combination therapy”, among others. Only peer-reviewed original research articles and reviews written in English were considered. The reference lists of retrieved articles were also manually screened to identify additional relevant studies. Given the narrative nature of this review, studies were selected based on their relevance to the conceptual framework of a spatiotemporally regulated PCD network in renal IRI. Emphasis was placed on emerging mechanisms and therapeutic strategies.

This review aims to synthesize existing knowledge by constructing a dynamic network model of the interactions among these novel PCD pathways in renal IRI, rather than merely cataloging them. We will first outline the core mechanisms and evidence for each death modality. We will then focus on their crosstalk and argue that the emergent properties of this network, rather than the sum of its parts, determine the magnitude of renal injury. Finally, we will critically evaluate therapeutic strategies targeting network nodes and propose a future research agenda. This agenda will focus on system-level approaches to modulate this decisive network for renal protection.

## 2 Novel PCD pathways in renal IRI: core mechanisms

### 2.1 Pyroptosis: the inflammatory executor

**Figure 1:**
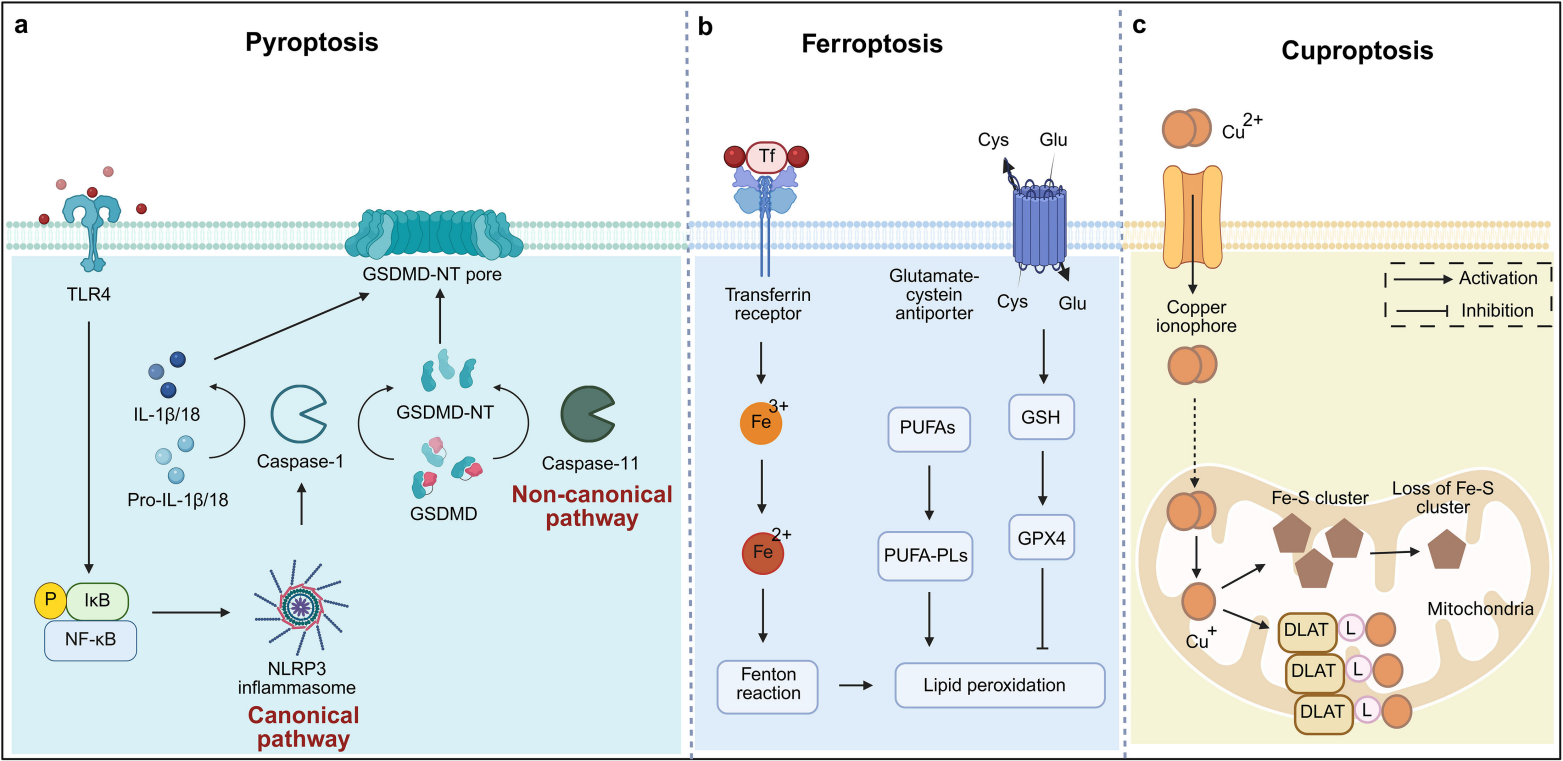
Mechanisms of pyroptosis, ferroptosis and cuproptosis. **(a)** Pyroptosis. Renal IRI triggers the assembly and activation of the NLRP3 inflammasome. Activated caspase-1 processes pro-IL-1β and pro-IL-18 into mature IL-1β and IL-18. It also cleaves GSDMD, releasing GSDMD-NT. GSDMD-NT oligomerizes and forms pores in the plasma membrane. Concurrently, renal IRI upregulates caspase-11 expression. Activated caspase-11 directly cleaves GSDMD, executing pyroptosis independently of the NLRP3 inflammasome. **(b)** Ferroptosis. Its core mechanism is driven by iron-dependent lipid peroxidation. Pathological stimuli, such as ischemia-reperfusion, lead to intracellular ROS accumulation and iron overload. These two factors act synergistically via the Fenton reaction. This reaction catalyzes the catastrophic accumulation of phospholipid hydroperoxides. The result is oxidative damage and functional loss of cellular membranes, especially mitochondrial membranes. The GSH-GPX4 antioxidant system serves as the core cellular defense against this process. Loss of its function, including GPX4 inactivation or GSH depletion, serves as the key switch that triggers ferroptosis. **(c)** Cuproptosis. The highly active Cu^+^ enters mitochondria. It directly attacks and disrupts the structure of Fe-S cluster proteins, leading to their disassembly. The loss of Fe-S clusters causes lipoylated enzymes, such as DLAT, to misfold, aggregate and lose function. This triggers severe mitochondrial proteotoxic stress, ultimately resulting in cell death.

Pyroptosis is a lytic and pro-inflammatory form of PCD mediated by Gasdermin family proteins, primarily Gasdermin D (GSDMD) (12). In renal IRI, its activation involves the coordinated action of both canonical and non-canonical inflammasome signaling pathways (**Figure 1a**). During renal IRI, the NLR family pyrin domain containing 3 (NLRP3) inflammasome is significantly activated in renal tissue. Activated caspase-1 cleaves GSDMD, releasing its N-terminal fragment. This fragment oligomerizes to form plasma membrane pores, leading to the release of potent pro-inflammatory cytokines, such as IL-1β and IL-18. These events are closely associated with renal tubular epithelial cell damage and the elevation of renal function markers, including Scr and BUN (13, 14). In addition to the canonical NLRP3/caspase-1 pathway, renal IRI also upregulates the expression of caspase-11, a component of the non-canonical pathway. Activated caspase-11 cleaves GSDMD, thereby triggering pyroptosis in renal tubular epithelial cells (15). Notably, Gasdermin E (GSDME) is highly expressed in renal tubules and can be cleaved by caspase-3. This process converts an apoptotic signal into a pyroptotic outcome, rendering GSDME a critical hub for the convergence of different cell death pathways (16).

### 2.2 Ferroptosis: a metabolic catastrophe

Ferroptosis is an iron-dependent form of cell death driven by the catastrophic accumulation of phospholipid hydroperoxides (17). Its core defense system is the glutathione (GSH)-glutathione peroxidase 4 (GPX4) axis. Depletion of GSH or inactivation of GPX4 abolishes the reduction of lipid hydroperoxides, leading to membrane damage (18–20). In the pathological progression of renal IRI, the activation of ferroptosis exhibits distinct spatiotemporal characteristics. During the ischemic phase, hypoxia leads to the accumulation of reactive oxygen species (ROS) and the release of iron ions, laying the metabolic groundwork for ferroptosis. The reperfusion phase, however, paradoxically triggers even more intense oxidative stress upon the restoration of oxygen supply. This, combined with iron overload, creates a synergistic effect. It initiates explosive lipid peroxidation through the Fenton reaction, a scenario aptly described as a “perfect storm” (21, 22) (**Figure 1b**). Renal tubular epithelial cells are highly sensitive to oxidative damage and consequently undergo widespread death. Their characteristic pathological hallmarks include mitochondrial shrinkage and loss of cristae, which serve as morphological evidence of ferroptosis. Ferroptosis is not a “silent” death. Dying cells release damage-associated molecular patterns (DAMPs). These DAMPs activate the innate immune system, recruit inflammatory cell infiltration and promote the activation of myofibroblasts as well as extracellular matrix deposition. This process further exacerbates renal interstitial inflammation and fibrosis following acute injury (23, 24). Importantly, ferroptosis can occur synchronously across multiple tubules, suggesting a coordinated and propagative mechanism of injury.

### 2.3 Cuproptosis: metal-induced proteotoxicity

Cuproptosis is a newly defined cell death pathway. It is triggered by excessive copper binding to lipoylated enzymes in the tricarboxylic acid (TCA) cycle. This binding leads to enzyme aggregation, functional loss and proteotoxic stress, ultimately culminating in cell death (25) (**Figure 1c**). Copper homeostasis is tightly regulated and its disruption is implicated in disease. Although recent studies have begun to elucidate the role of cuproptosis in renal IRI, the evidence remains preliminary and requires further validation. At this stage, cuproptosis should be regarded as a highly promising emerging hypothesis, avoiding equating its research maturity entirely with that of ferroptosis or pyroptosis. Emerging evidence suggests that cuproptosis may occur in renal tubular epithelial cells following renal IRI (26). Preliminary observations indicate that it may manifest as cellular edema, nuclear pyknosis and functional impairment. Prolonged or severe cuproptosis could further lead to tubular atrophy and interstitial fibrosis, thereby exacerbating structural damage to kidney tissue (26). Iron overload may increase cellular susceptibility to cuproptosis, possibly by disrupting iron-sulfur cluster biogenesis (27). This finding suggests a potential mechanism that dysregulation of iron homeostasis might serve as a critical bridge linking ferroptosis and cuproptosis. These are two distinct metal-associated cell death modalities. The morphological characteristics and functional consequences of cuproptosis position it as a relevant yet currently underexplored contributor to renal IRI.

### 2.4 Other key players: the contextual dual roles of necroptosis and autophagy

Necroptosis is a programmed form of necrosis activated upon inhibition of apoptosis, for instance when caspase activity is blocked. It is initiated by death receptors or pattern recognition receptors (28). The activation of this pathway is caspase-independent and is instead driven by a signaling cascade that involves RIPK1, RIPK3 and their common substrate MLKL (29). In renal IRI, signals such as hypoxia and oxidative stress can significantly upregulate the activity of key molecules in the necroptotic pathway, including RIPK1, RIPK3 and MLKL. This cascade mediates the programmed rupture of renal epithelial cells. This rupture amplifies local inflammation through the release of DAMPs, thereby exacerbating renal function impairment (30, 31).

Autophagy, as one of the most extensively studied traditional cell death modalities, exhibits a context-dependent role in renal IRI. The protective or detrimental function of autophagy depends primarily on the timing and intensity of its activation. During the ischemic phase, moderate autophagy can clear damaged mitochondria and oxidized proteins. This provides energy and substrates for cell survival, thus exerting a protective effect. However, during the reperfusion phase, excessive or dysregulated autophagy may non-selectively degrade essential cellular components or engage in crosstalk with apoptotic signals. It can then transform into a factor that promotes cell death and aggravates tissue damage (32). Key determinants of this shift include the duration of ischemia, the phase of the IRI process and the extent of autophagic flux (33).

## 3 The integrated PCD network: crosstalk as a core driver of injury

Cell death pathways do not operate in isolation. Instead, they are interconnected through a complex molecular network that forms a sophisticated system of cross-regulation. This interaction manifests mainly at three levels. Different death pathways share key regulatory molecules. One example is the dual role of the caspase family in both apoptosis and pyroptosis. There is also a typical sequential relay pattern. In this pattern, the inhibition of apoptosis can shift cellular fate towards necroptosis. Moreover, different death modalities can transform into one another. For instance, the ferroptosis inducer Erastin can simultaneously suppress apoptotic pathways. This intricate network of interactions is of significant importance in the pathogenesis, progression and treatment of diseases. It provides a new theoretical foundation for developing combination therapies for complex diseases.

### 3.1 The inflammatory axis: pyroptosis and other death modalities fuel a vicious cycle

Pyroptosis and necroptosis are co-activated and engage in bidirectional amplification. This creates a potent inflammatory microenvironment. They share common triggers and molecular bridges. DAMPs released from damaged cells simultaneously activate the RIPK1/RIPK3 cascade and the toll-like receptor 4 (TLR4)/nuclear factor kappa-B (NF-κB) pathway, which primes NLRP3 inflammasome assembly. Crucially, the necroptosis executioner protein MLKL can promote NLRP3 inflammasome activation and IL-1β maturation independently of GSDMD (34, 35). Under physiological conditions, GSDMD-mediated pyroptosis leads to rapid cell lysis. This effectively preempts the slower activation of the necroptosis and thereby exerts a natural inhibitory effect. However, in renal IRI, this inhibition is abolished when GSDMD function is impaired or its expression is insufficient. Consequently, renal tubular epithelial cells and macrophages become significantly more susceptible to necroptosis (36). This establishes a positive feedback loop. Cell lysis caused by necroptosis releases DAMPs, which activate inflammasomes in neighboring cells. This leads to pyroptosis and cytokine release. These cytokines create a pro-inflammatory microenvironment that further sensitize cells to TNFα-mediated necroptosis, driving uncontrolled inflammation and exacerbated tissue damage. These findings reveal experimentally validated functional interactions between pyroptosis and necroptosis, as documented by MLKL-dependent NLRP3 activation and GSDMD-mediated preemption of necroptosis.

**Figure 2:**
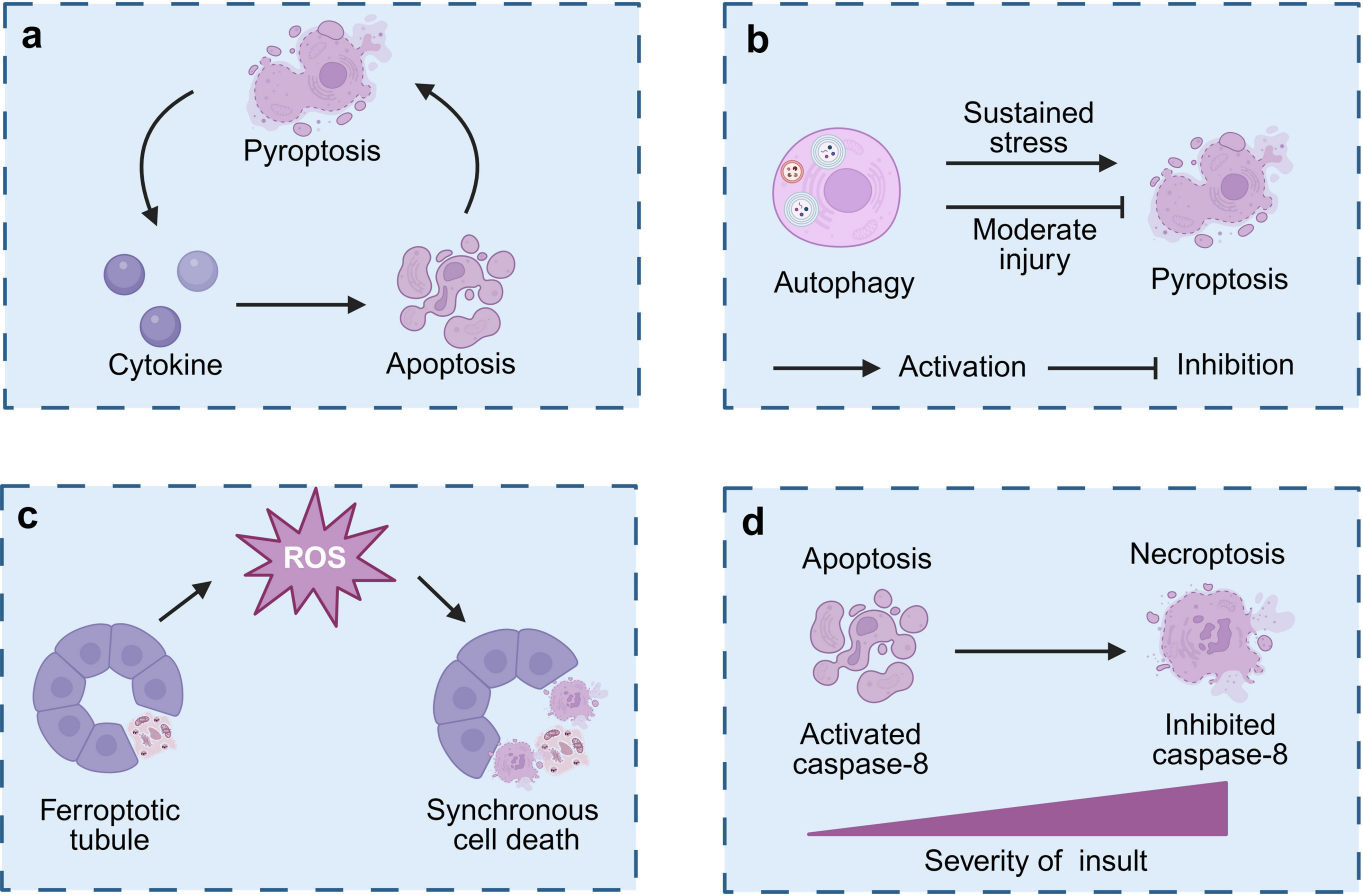
Interactions between different cell death modalities. **(a)** Inflammatory factors released from pyroptotic cell lysis exacerbate local inflammation and oxidative stress. This microenvironment in turn promotes apoptosis in more cells. Simultaneously, under specific conditions, apoptotic signals can be converted into pyroptosis. Damage-associated molecules released by both modalities further activate immune responses. This forms a self-reinforcing positive feedback loop that continuously amplifies tissue damage and cell death. **(b)** The regulation of pyroptosis by autophagy exhibits a complex “double-edged sword” characteristic. Autophagy suppresses the initiation and execution of pyroptosis by clearing damaged mitochondria and selectively degrading key activated pyroptosis-related proteins. However, under severe stress, excessive or dysregulated autophagy fails to effectively clear damage signals. Instead, it switches to promoting pyroptosis. **(c)** The death of renal tubular epithelial cells exhibits a marked spatially synchronized pattern. Ferroptosis is a key mechanism underlying this pathological phenomenon. When the local tissue’s redox defense system collapses, signals such as peroxidized lipids released from damaged cells can diffuse among neighboring cells. This creates a self-amplifying wave of damage, driving clusters of cells to synchronously enter an irreversible death program. Consequently, this causes continuous destruction of the tubular structure. **(d)** Cell death displays a temporally biphasic pattern. The first wave is directly triggered by the initial insult, primarily manifesting as apoptosis or acute necrosis. Subsequently, factors persistently present in the microenvironment activate death receptor pathways. This initiates a delayed but more extensive second wave of death. This phase is dominated by necroptosis. Its executioner protein MLKL mediates membrane damage and releases pro-inflammatory substances. This exacerbates oxidative stress and local inflammation. The fate decision between apoptosis and necroptosis is primarily determined by the activity status of caspase-8.

ROS serve as the core initiating signal for both pyroptosis and apoptosis. In renal IRI, ROS activate the NLRP3 inflammasome, directly inducing pyroptosis. Subsequently, IL-1β and IL-18 exacerbates inflammation and oxidative stress. This creats a positive feedback loop that amplifies tissue damage and promotes further cell death (**Figure 2a**). Concurrently, ROS induce mitochondrial membrane potential collapse and cytochrome C release. This activates the caspase-9/caspase-3 cascade to execute apoptosis (37). Furthermore, in hypoxia- and ischemia-induced neuronal injury, pyroptosis can activate apoptosis-related proteins (38). The canonical pyroptosis pathway may shift towards apoptosis (39). The non-canonical pathway can also indirectly induce apoptosis (40). Conversely, apoptosis effectors can promote NLRP3 inflammasome activation via the cellular FLICE-like inhibitory protein (c-FLIP) complex or the NF-κB pathway (41, 42). FAS-associated death domain protein (FADD) also promotes the activation of the NLRP3 inflammasome and caspase-1, thereby regulatig pyroptosis (43). Notably, GSDME is highly expressed in renal tubular epithelial cells. In these cells, caspase-3 cleaves GSDME, thereby converting apoptosis into pyroptosis (44). Conversely, caspase-3 may also inhibit pyroptosis by cleaving activated GSDMD-NT (45), indicating bidirectional regulation. This body of evidence confirms functional crosstalk between pyroptosis and apoptosis, as shown by FADD-dependent NLRP3 activation and caspase-3-mediated GSDME cleavage. This dynamic network enables signal conversion and amplification, significantly expanding the scope and intensity of injury.

Pyroptosis and autophagy form a complex “double-edged sword” network, collectively determining cell fate. Typically, autophagy is cytoprotective by clearing damaged mitochondria and abnormal protein aggregates. In contrast, pyroptosis causes plasma membrane rupture and amplifies inflammation via IL-1β (46). In various kidney injury diseases, autophagy inhibits pyroptosis at multiple levels (47–49). Autophagy clears damaged mitochondria. This reduces the production of mitochondrial ROS (mtROS), which is a key trigger for NLRP3 inflammasome activation (50). Furthermore, autophagy degrades components of the activated NLRP3 inflammasome. For instance, sequestosome 1 (P62) mediates the degradation of apoptosis-associated speck-like protein containing a CARD (ASC) specks (51). Tripartite motif containing 20 (TRIM20) targets both NLRP3 and pro-caspase-1 (52). Ubiquitin specific peptidase 18 (USP18) mediates ubiquitination of GSDMD, subsequently mediating its degradation (53). Additional, immunity-related GTPase family M protein (IRGM), ubiquitin specific peptidase 5 (USP5) and galectin-9 (LGALS9) similarly regulate pyroptosis through analogous mechanisms (54–56). However, under IRI stress, excessive autophagy fails to provide protection and instead depletes cellular components. It paradoxically amplifies pyroptotic signaling. Conversely, pyroptosis driven inflammation further disrupts autophagic homeostasis, creating a positive feedback loop (57–59) (**Figure 2b**). Moreover, in AKI, autophagy activates caspase-3 and caspase-8, which then drive GSDME-mediated pyroptosis (60, 61). These findings establish experimentally supported crosstalk between pyroptosis and autophagy, as reflected by autophagy-mediated degradation of NLRP3 inflammasome components, as well as by autophagy-dependent activation of the caspase-3/caspase-8 axis that drives GSDME-mediated pyroptosis.

In renal IRI, pyroptosis and ferroptosis constitute a mutually reinforcing positive feedback loop. This loop collectively exacerbates the loss of renal tubular epithelial cells and the deterioration of renal function (62–64). In neonicotinoid-induced kidney injury, ferroptosis releases high mobility group protein B1 (HMGB1). HMGB1 then activates the HMGB1-receptor for advanced glycosylation end products (RAGE)/TLR4/NF-κB signaling pathway to trigger pyroptosis (65). Furthermore, free iron accumulated during ferroptosis catalyzes the production of ROS via the Fenton reaction. In diquat-induced kidney injury, these ROS further activate caspase-3, which then cleaves GSDME to promote pyroptosis (66). On the other hand, pyroptosis releases inflammatory cytokines. These cytokines weaken antioxidant defense system and amplify oxidative stress, further promoting ferroptosis. Within this regulatory network, GPX4 acts as a key regulatory factor. In cadmium-induced nephrotoxicity, GPX4 directly inhibits ferroptosis by reducing phospholipid hydroperoxides. Simultaneously, by maintaining mitochondrial function and reducing mtROS generation, GPX4 indirectly inhibits NLRP3 inflammasome activation (67). The nuclear factor erythroid 2-related factor 2 (Nrf2)/kelch-like ECH-associated protein 1 (KEAP1) axis upregulates heme oxygenase-1 (HO-1) and GPX4, promoting GSH synthesis and inhibiting ferroptosis (68, 69). In osteoarthritis, Nrf2 also exhibits a dual function in pyroptosis. It can promote pyroptosis by activating the NLRP3 and absent in melanoma 2 (AIM2) inflammasomes, yet it can also inhibit pyroptosis by reducing intracellular ROS levels (70). Overall, these findings substantiate experimentally supported crosstalk between pyroptosis and ferroptosis, as illustrated by ferroptosis-derived HMGB1 and ROS that trigger pyroptosis, as well as shared regulatory nodes including Nrf2 that bidirectionally modulate both pathways.

### 3.2 The metabolic stress axis: ferroptosis and other cell death modalities induce metabolic imbalance

In renal injury, autophagy serves as a crucial upstream regulator of ferroptosis, forming a complex bidirectional regulatory network (71). In AKI, chaperone-mediated autophagy degrades GPX4, ultimately triggering ferroptosis (72, 73). Various forms of selective autophagy also regulate ferroptosis through distinct mechanisms (74). In IRI, ferritinophagy releases iron ions into the cytosol. This in turn catalyzes ROS production via the Fenton reaction, ultimately induces ferroptosis (75). In cisplatin-induced nephrotoxicity, mitophagy reduces ROS levels, leading to a decrease in HO-1. The decline in HO-1 alleviates its inhibitory effect on GPX4, thereby inhibiting ferroptosis (76). Concurrently, lipophagy promotes lipid peroxidation by releasing fatty acids (77, 78). In hepatocellular carcinoma, reticulophagy inhibits ferroptosis by alleviating endoplasmic reticulum (ER) stress (79, 80). In neuronal and intervertebral disc degeneration, lysophagy inhibits ferroptosis by maintaining iron metabolic homeostasis (81, 82), yet it can also promote ferroptosis by degrading GPX4 (83). Acyl-CoA synthetase long-chain-fatty-acid--CoA ligase 4 (ACSL4) is a key promoter of ferroptosis. It drives ferroptotic by mediating the formation of lipid peroxidation substrates (84). In addition, ACSL4 influences autophagy via the protein kinase AMP-activated catalytic subunit alpha 1 (AMPK)/mammalian target of rapamycin (mTOR) signaling pathways and impedes autophagosome formation by inhibiting proton pump function (85, 86). Conversely, in diabetic retinopathy, the degradation of ACSL4 via the chaperone-mediated autophagy pathway can effectively inhibit ferroptosis (87). These findings experimentally validate functional crosstalk between autophagy and ferroptosis, as demonstrated by chaperone-mediated GPX4 degradation, ferritinophagy-driven iron release, mitophagy-mediated ROS reduction, lipophagy-promoted lipid peroxidation, reticulophagy-mediated ER stress alleviation, lysophagy-dependent iron homeostasis and ACSL4-dependent autophagy regulation.

In sepsis-induced AKI, ferroptosis and apoptosis form a synergistic amplification network through shared mitochondrial damage and oxidative stress (88). Apoptosis induced mitochondrial outer membrane permeabilization (MOMP) releases cytochrome c and dysregulates iron metabolism. This promotes the Fenton reaction, accelerates lipid peroxidation and facilitates the transition from apoptosis to ferroptosis. More critically, a positive feedback loop exists between the two pathways. ROS generated during the execution phase of apoptosis further inactivates GPX4. Conversely, lipid peroxides accumulated during ferroptosis exacerbates mitochondrial damage, thereby accelerating apoptosis. In cancer, ferroptosis induces ER stress and the unfolded protein response (UPR). This activates the DNA damage inducible transcript 3 (CHOP)/p53-upregulated modulator of apoptosis (PUMA) axis to initiate apoptosis (89). Conversely, cellular tumor antigen p53 (P53) exerts dual regulation on ferroptosis. It promotes ferroptosis by suppressing the function of solute carrier family 7 member 11 (SLC7A11) and GPX4. On the other hand, it inhibits ferroptosis by interacting with dipeptidyl peptidase IV (DPP4) (90, 91). ACSL4 serves as a crucial molecular hub connecting ferroptosis and apoptosis. It promotes fatty acyl-CoA generation, enhancing oxidative stress and activating the intrinsic apoptotic pathway (92, 93). However, in cancer, during ferroptosis, fatty acid oxidation-induced signal transducer and activator of transcription 3 (STAT3) upregulates ACSL4 expression, which paradoxically maintains mitochondrial integrity and suppresses apoptosis (94). Synthesizing these findings, experimental evidence supports functional crosstalk between ferroptosis and apoptosis, as indicated by MOMP-driven iron dysregulation that promotes ferroptosis, CHOP-PUMA axis activation by ferroptosis that initiates apoptosis, p53-dependent bidirectional regulation of ferroptosis and ACSL4 as a molecular hub connecting both pathways.

In renal injury, the death of renal tubular epithelial cells exhibits a synchronized pattern, with ferroptosis as a primary driver. Within a localized area, once the antioxidant defense system collapses, adjacent cells enter an irreversible death program almost simultaneously (**Figure 2c**). Notably, ferroptosis and necroptosis form a complex bidirectional network. Ferroptosis derived lipid peroxides activate the RIPK1/RIPK3/MLKL pathway, thereby amplifying necroptosis. On the other hand, MLKL causes potassium efflux, activating the NLRP3 inflammasome and creating a pro-oxidative stress microenvironment conducive to ferroptosis (95). In addition, ferroptosis can also inhibit necroptosis. Ferroptosis pores trigger calcium influx, activating the endosomal sorting complexes required for transport (ESCRT)-III complex. The ESCRT-III complex repairs membrane pores to slow ferroptosis and inhibit MLKL-mediated necroptosis (96). In acute kidney failure, ACSL4 bridges the two death pathways. Certain lipid products it generates create an environment favorable for MLKL oligomerization and membrane pore formation (97). Furthermore, cysteine metabolism plays a central role in their cross-regulation. On one hand, mtROS drive the autophosphorylation of RIPK1, thereby initiating necroptosis (98). On the other hand, cysteine promotes GSH synthesis, which in turn inhibits ferroptosis (99). In melanoma, blocking this system leads to ROS accumulation, simultaneously inducing both death modalities (100). The molecular chaperone heat shock protein 90 homolog (HSP90) plays a dual role in this network. In neurons, HSP90 promotes the formation of the RIPK1/RIPK3 necrosome complex, thereby facilitating necroptosis (101). It can also mediate the degradation of GPX4, activating ferroptosis (102). More importantly, in cerebral IRI, HSP90 overexpression can simultaneously activate both death pathways via the HSP90–GCN2–ATF4 signaling axis. GCN2 denotes the eIF-2-alpha kinase and ATF4 stands for activating transcription factor 4 (103). Additionally, in osteoblastic cells, iron overload also promotes necroptosis by elevating ROS levels (104). The above findings demonstrate experimentally supported interplay between ferroptosis and necroptosis, as manifested by ferroptosis-derived lipid peroxides that activate RIPK1/RIPK3/MLKL, MLKL-driven potassium efflux that promotes ferroptosis, ESCRT-III-mediated membrane repair that inhibits necroptosis, ACSL4-generated lipid products that facilitate MLKL oligomerization, cysteine metabolism blockade and the HSP90-GCN2-ATF4 axis that simultaneously activates both pathways.

In cisplatin-induced AKI, ferroptosis and cuproptosis do not occur in isolation. Instead, they promote each other through shared axes of metabolic imbalance and oxidative stress, forming a synergistically amplified injury network. Ferroptosis exacerbates mitochondrial damage through lipid peroxidation, which in turn promotes cuproptosis. Conversely, mitochondrial collapse induced by cuproptosis further weakens cellular antioxidant capacity, thereby accelerating ferroptosis. Iron overload downregulates proteins involved in iron-sulfur cluster biogenesis, including the iron-sulfur cluster assembly enzyme (ISCU) and the HIRA-interacting protein 5 (NFU1). Iron-sulfur clusters serve as essential cofactors for numerous mitochondrial enzymes, including those involved in copper metabolism. The destabilization of these clusters increases susceptibility to cuproptosis, rendering mitochondria more vulnerable to copper-induced protein aggregation. Mitochondrial dysfunction serves as a common hub for both ferroptosis and cuproptosis. The hallmarks of ferroptosis include mitochondrial cristae reduction and increased membrane density, with GPX4 inactivation leading to oxidative damage of the mitochondrial membrane. Cuproptosis, on the other hand, directly targets mitochondrial TCA cycle enzymes, causing their aggregation and subsequent respiratory chain collapse (105). Collectively, the data support functional crosstalk between ferroptosis and cuproptosis, as revealed by iron overload-induced destabilization of iron-sulfur clusters that sensitizes tubular cells to cuproptosis and mitochondrial dysfunction as a shared hub for both pathways.

### 3.3 Interactions between other cell death modalities

Autophagy and necroptosis maintain a dynamic, mutually restrictive equilibrium. Moderate autophagy degrades damaged mitochondria and oxidative stress products, indirectly suppressing necroptosis. Furthermore, autophagosomes directly engulf and degrade RIPK1 and RIPK3, negatively regulating this pathway. However, excessive autophagic flux engages in crosstalk with necroptotic signals. It then exhibits a pro-death phenotype that synergistically exacerbates tubular damage. Notably, once necroptosis is activated, MLKL-mediated membrane rupture triggers inflammation and disrupts the integrity of autophagic flux, pushing it toward a destructive state. Necrotic cells also recruit inflammatory cells and release DAMPs, persistently activating death receptor pathways. Ultimately, this forms a vicious cycle of “autophagy-necroptosis-inflammation”, collectively driving renal injury (106–108). RIPK3 mediates the phosphorylation and activation of AMPK. Activated AMPK enhances the activity of unc-51-like kinase 1 (ULK1) and beclin-1, thereby promoting autophagy (109). Furthermore, in cardiac IRI, phosphorylation of RIPK activates calcium/calmodulin-dependent protein kinase (CaMKII), which induces the opening of the mitochondrial permeability transition pore (MPTP) and triggers mitophagy via the RIPK3-PGAM5-Drp1 signaling axis. Here, PGAM5 stands for phosphoglycerate mutase family member 5 and Drp1 stands for density-regulated protein (110, 111). Conversely, in cisplatin-induced nephrotoxicity, ROS positively regulate necroptosis by inducing MPTP opening and activating the RIPK1/RIPK3/MLKL signaling pathway (112). They can also promote the autophosphorylation of RIPK1, enhancing its interaction with RIPK3 and thereby facilitating the assembly of the necrosome complex (113). Taken as a whole, these findings experimentally substantiate functional crosstalk between autophagy and necroptosis, as evidenced by RIPK3-mediated autophagy activation and autophagy-derived ROS that positively regulate necroptosis.

In renal IRI, apoptosis and autophagy are tightly coupled through oxidative stress and inflammatory responses. Their interaction exhibits a time- and dose-dependent dynamic evolution (114–116). During the early stage, autophagy exerts a protective, antagonistic effect. It degrades caspase-8, thereby blocking extrinsic apoptotic signaling. In addition, autophagy suppresses cytochrome c release and blocks the intrinsic apoptotic pathway (117). However, as injury progresses, autophagy transforms into a pro-apoptotic synergistic mechanism. P62-mediated degradation of Fas-associated protein-tyrosine phosphatase 1 (Fap-1) enhances Fas phosphorylation, increasing cellular sensitivity to apoptosis (118). Autophagosomes engulf mitochondria to maintain the immunologically silent state of apoptosis (119). Autophagy-related protein 12 (ATG12) inhibits the anti-apoptotic proteins B-cell lymphoma 2 (Bcl-2) and myeloid cell leukemia 1 (Mcl-1), thereby activating BCL2 associated X protein (BAX) (120). Additionally, truncated autophagy protein 5 (ATG5) alters MOMP and activates the caspase cascade (121). Concurrently, in ovarian cancer, apoptotic effector caspases, such as caspase-3, -6, -7, -8, and -9, specifically cleave Beclin-1 and autophagy-related protein 3 (ATG3). This disrupts the integrity of autophagic flux (122, 123). Notably, in renal IRI, B-cell lymphoma extra-large (Bcl-xL) interacts with Beclin-1 to constitutively inhibit autophagosome formation. Simultaneously, by stabilizing the mitochondria, it prevents cytochrome c release. Changes in its expression levels influence the balance between autophagy and apoptosis, ultimately guiding the dynamic progression from mutual antagonism to synergistic promotion (124). The evidence presented here supports functional crosstalk between apoptosis and autophagy, as observed in autophagy-driven apoptosis via P62/Fap-1, ATG12/Bcl-2/Mcl-1 and truncated ATG5, caspase-mediated disruption of autophagy, and Bcl-xL as a key regulatory node balancing both pathways.

Apoptosis and necroptosis interact across different stages, collectively driving the progression and amplification of injury. In renal IRI, cell death exhibits a temporally distinct biphasic pattern. The first wave is directly triggered by the initial insult, including ischemia or toxins, and primarily manifests as apoptosis or acute necrosis. Subsequently, cytokines, such as TNF-related weak inducer of apoptosis (TWEAK), persistently activate death receptor pathways. This initiates a delayed but more extensive second wave. During this second wave, necroptosis acts as an upstream event. MLKL mediated membrane rupture releases DAMPs. These DAMPs exacerbate oxidative stress and inflammatory responses, thereby significantly promoting apoptosis. The two death modalities act synergistically, jointly driving a vicious cycle that leads to the continuous escalation of tissue damage (125–127) (**Figure 2d**). The cellular fate is precisely regulated by caspase-8. When caspase-8 actives, it initiates apoptosis by activating caspase-3. At the same time, it inhibits necroptosis by cleaving RIPK1, RIPK3 and cylindromatosis (CYLD) (128, 129). Conversely, when caspase-8 activity is inhibited, the cell shifts towards necroptosis (130). Beyond caspase-8, RIPK1 and RIPK3 also participate in the regulation of apoptosis. Within the tumor necrosis factor (TNF) receptor signaling pathway, loss of RIPK3 kinase activity promotes apoptosis through a switch in the function of adaptor proteins (131). This network of interactions is likely a core mechanism underlying the progression from acute to chronic injury accompanied by uncontrolled inflammation in renal IRI. In aggregate, these findings validate experimentally supported crosstalk between apoptosis and necroptosis, as underscored by caspase-8 serving as a molecular switch and loss of RIPK3 kinase activity that promotes apoptosis.

Based on the evidence reviewed above, a tentative hierarchy within the PCD network in renal IRI can be proposed. Mitochondrial dysfunction and ROS production serve as the central initiating hub at the apex of the hierarchy. Downstream, the network branches into parallel but interconnected pathways. Necroptosis and pyroptosis appear to dominate during the early inflammatory phase. Ferroptosis becomes increasingly prominent during the reperfusion phase, when iron accumulation and lipid peroxidation peak. Apoptosis may represent a default pathway that is engaged when other routes are blocked. Cuproptosis remains the least characterized component. Its position in the hierarchy may be context-dependent rather than fixed. Autophagy occupies a unique position, functioning predominantly as a negative regulator at baseline but potentially switching to a pro-death role under extreme stress. Importantly, this proposed hierarchy is not static. The relative contribution of each pathway likely shifts over time and varies across cell types.

### 3.4 Limitations and uncertainties in the current PCD network model

Despite growing evidence supporting a coordinated PCD network in renal IRI, several important limitations and uncertainties must be acknowledged. Translational relevance from preclinical models to human disease remains uncertain and clinical validation is lacking. Most studies have been conducted in rodent models. It remains unclear how faithfully these findings translate to human renal IRI. Species differences in the expression and regulation of key cell death mediators, such as GSDMD, MLKL and GPX4, may limit direct extrapolation. To date, no therapy targeting the PCD network has been approved specifically for renal IRI. The safety of long-term modulation of these pathways in humans also remains unknown. Spatiotemporal dynamics and cell type-specificity of the network are poorly defined. Most studies examine a single time point This makes it difficult to capture the dynamic, time-dependent nature of the network. The temporal sequence of activation of different PCD pathways and the critical time windows for therapeutic intervention remain unknown. Furthermore, nearly all data come from bulk tissue analysis, which cannot distinguish cell type-specific contributions. The PCD network likely operates differently in proximal tubular epithelial cells, distal tubular cells, endothelial cells and infiltrating immune cells. In contrast to the well documented crosstalk of pyroptosis and ferroptosis, the interplay of cuproptosis with other PCD modalities remains the least characterized. To date, only the connection between cuproptosis and ferroptosis has been explored. Its interactions with pyroptosis, necroptosis and autophagy have yet to be fully elucidated. Further studies are therefore warranted to define the relative contribution of cuproptosis within the broader PCD network in renal IRI. The functional consequences of pathway redundancy and compensation are poorly understood. When one death pathway is inhibited, it is unclear whether other pathways reliably compensate.It is also unclear whether such compensation is beneficial or harmful. This knowledge gap has significant implications for the development of combination therapies.

Addressing these limitations will require refined animal models that better recapitulate human disease. It will also require dynamic monitoring of pathway activation using novel biomarkers and single-cell multi-omics approaches to resolve cell type-specific network architecture. Despite these uncertainties, the framework of a PCD network provides a valuable conceptual foundation for understanding the complexity of renal IRI and for developing next-generation therapeutic strategies.

### 3.5 Broader implications: PCD networks in other kidney diseases

While this review focuses on renal IRI, the concept of an integrated PCD network is also relevant to other common kidney diseases, such as chronic kidney disease (CKD) and diabetic kidney disease (DKD). Understanding how these networks operate in different disease contexts may reveal shared pathophysiological mechanisms and disease-specific therapeutic opportunities.

Emerging evidence suggests that similar PCD networks occur in CKD. GSDMD mediated pyroptosis plays a protective role by suppressing necroptosis. Moreover, the balance between these two pathways determines the outcome of CKD (132). Autophagy negatively regulates pyroptosis by clearing damaged mitochondria and limiting ROS driven inflammasome activation, thereby alleviating 3-MCPD-induced renal injury (133). Cuproptosis enhances ferroptosis by upregulating substrate for lipid peroxidation, such as PE, and by damaging the antioxidant protein GPX4 (134). Furthermore, restoration of autophagy and suppression of apoptosis act synergistically to ameliorate intestinal barrier dysfunction in CKD (135). In some contexts, different PCD pathways act cooperatively rather than antagonistically. In a rat model of CKD, both necroptosis and apoptosis contribute to the progressive loss of renal tubular cells. Notably, necroptosis predominates in driving tubular cell loss and CKD progression (136). Moreover, PANoptosis in CKD operates as a flexible “death signaling network” that integrates key molecular components from pyroptosis, apoptosis and necroptosis. This network is a major contributor to renal tubular epithelial cell injury and the progression of interstitial fibrosis. The underlying mechanisms involve inflammation, oxidative stress and fibrotic processes. Several PANoptosis-related genes, such as prostaglandin G/H synthase 2 (PTGS2) and protein c-Fos (FOS), show abnormal expression patterns. PTGS2 is upregulated, where it may drive epithelial-mesenchymal transition (EMT) and accelerate renal fibrosis via the TGF-β/SMAD family member 3 (Smad3) axis (137). In addition, overexpression of FOS positively correlates with tubular atrophy and inflammatory infiltration in CKD models. Notably, certain traditional Chinese medicine formulas have been shown to slow CKD progression by downregulating FOS expression, possibly through interrupting the activator protein 1 (AP-1)/TNF-α positive feedback loop (138). The PCD network in CKD is characterized by multifaceted crosstalk. Pyroptosis and autophagy exert either protective or detrimental effects depending on the context, whereas cuproptosis and ferroptosis amplify injury, as well as necroptosis drives tubular cell loss. Meanwhile, PANoptosis primarily promotes tubular injury and interstitial fibrosis. This complex regulatory architecture highlights the importance of context-dependent modulation of cell death pathways for therapeutic intervention in CKD.

Accumulating evidence indicates that similar PCD networks operate in DKD. Their intricate interplay and mutual modulation have been increasingly recognized. Pyroptosis and ferroptosis act synergistically to promote the progression of DKD. Moreover, these two forms of cell death share common upstream regulators, namely mitochondrial damage and ROS (139). Similarly, ferroptosis and apoptosis cooperate to exacerbate podocyte injury and inflammation in DKD (140). In DKD, autophagy negatively regulates ferroptosis, as demonstrated by decreased lipid peroxidation and free iron accumulation, as well as increased levels of GPX4 and SLC7A11 (141, 142). An inverse relationship exists between autophagy and apoptosis. Thus, promoting autophagy can suppresses apoptosis, and this reciprocal regulation contributes to delaying DKD progression (143, 144). In DKD, PANoptosis is activated through the tumor necrosis factor ligand superfamily member 10 (TRAIL)/tumor necrosis factor receptor superfamily member 10B (DR5) signaling axis. Upon binding to its death receptor DR5, TRAIL triggers the assembly of a PANoptosome complex containing caspase-3, caspase-1 and MLKL. This complex simultaneously initiates apoptosis, pyroptosis and necroptosis. This integrated activation of multiple cell death pathways disrupts podocyte membrane integrity, releases pro-inflammatory mediators and promotes glomerulosclerosis. The activation level of the TRAIL/DR5 axis in podocytes positively correlates with disease severity in DKD patients (145). Although exogenous TRAIL administration improves renal function as well as glomerular and tubular morphology in diabetic mouse models, it has little effect on proteinuria (146). Collectively, the PCD network in DKD is characterized by synergistic interactions among pyroptosis, ferroptosis and apoptosis, which collectively drive disease progression. Autophagy serves as a protective counterbalance by negatively regulating both ferroptosis and apoptosis. Additionally, PANoptosis, activated via the TRAIL/DR5 axis, integrates multiple cell death pathways to exacerbate podocyte injury and glomerulosclerosis. Modulating the key regulators that maintain the balance among these interconnected cell death pathways may provide new therapeutic approaches for DKD.

In summary, integrated PCD networks are a conserved pathophysiological feature across renal IRI, CKD and DKD. While core crosstalk mechanisms are shared, disease-specific triggers, cell types and temporal patterns exist. Understanding these shared and distinct features is critical for developing tailored therapies. The interconnected nature of these pathways suggests that optimal outcomes may require network-based interventions rather than single-pathway inhibition.

## 4 Therapeutic strategies targeting the PCD network

**Figure 3:**
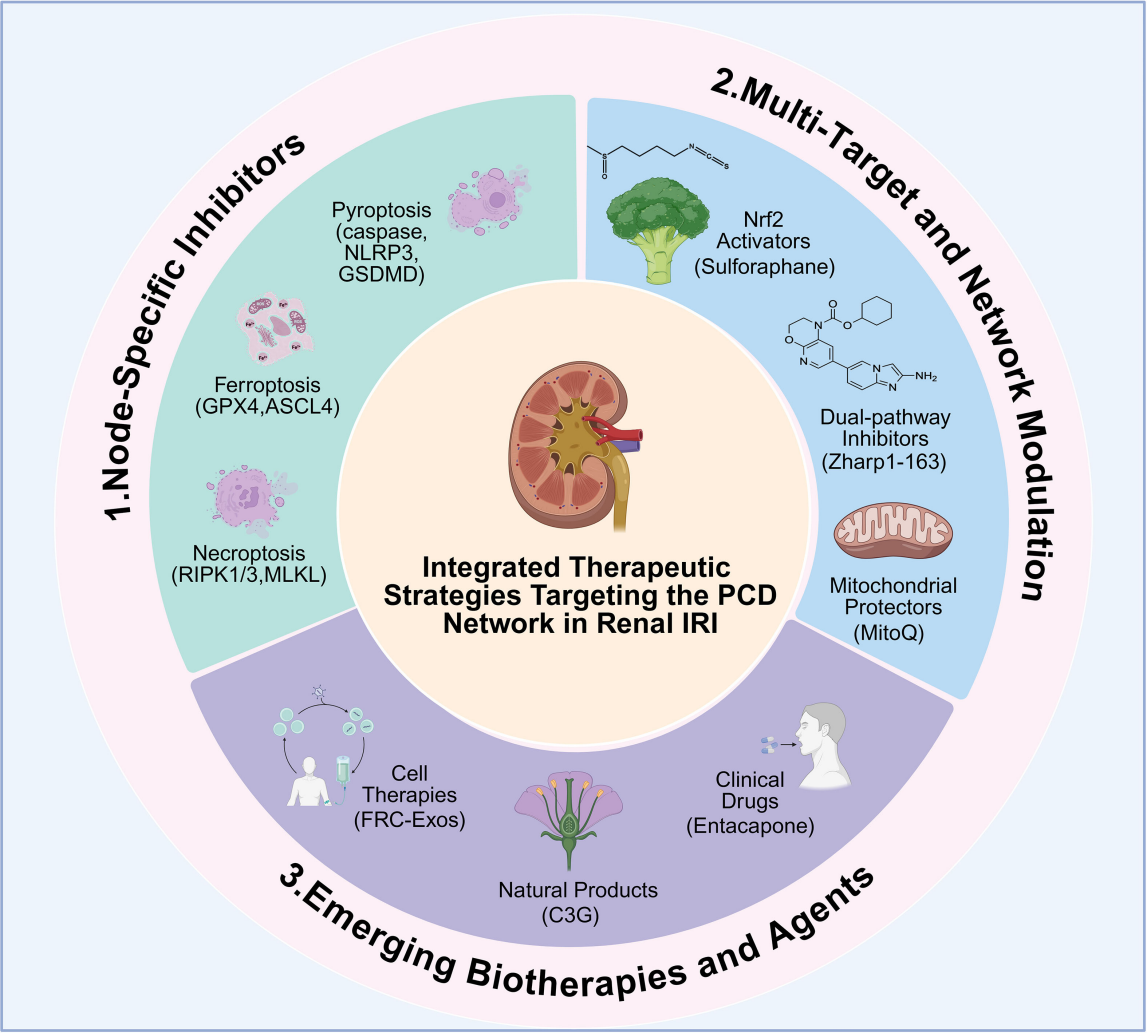
Integrated therapeutic strategies targeting the PCD network. Node-Specific Inhibitors. These inhibitors target key molecules of single pathways, including NLRP3, GPX4 and RIPK1. They represent the strategy with the broadest current research foundation and the most concentrated body of evidence. Multi-Target and Network Modulation Approaches. This core direction for transitioning to systemic intervention primarily includes upstream defense activation, for example Nrf2 activators, multi‑target drugs or combination therapies, such as dual‑pathway inhibitors, and shared stressor modulation, for instance mitochondrial protectors. This area is a key growth field for breaking through the current efficacy bottleneck. Emerging Biotherapies and Agents. This encompasses biological agents based on exosomes, nucleic acid drugs, cell therapies, multi-effective natural products and the repurposing of existing clinical drugs. This represents the most forward-looking exploratory field. These approaches focus on systemic regulation and intelligent delivery, providing new dimensions for future therapies.

Traditional drug development follows the linear paradigm of “one gene, one target, one disease”. It has achieved preliminary success in the early exploration of renal IRI, leading to the generation of various inhibitors that target key nodes of specific cell death pathways. However, the challenges encountered in clinical translation have unveiled a deeper pathological reality. The cell death network is characterized by redundancy, compensation and dynamic evolution. Consequently, therapeutic strategies must adapt accordingly. They should shift from attempting to “precisely destroy a single stronghold” toward “systematically regulating the entire battlefield”. We begin by reviewing the achievements of node-specific inhibitors and their inherent “compensation dilemma”. Subsequently, we focus on elucidating the next-generation strategies designed to overcome these limitations, including multi-target and network modulation approaches. Finally, we explore the frontier directions of utilizing emerging biotherapies and agents for the systematic remodeling of the death network (**Figure 3**). Currently, the majority of these drugs are still at the preclinical stage (**Table 1**). Together, these strategies form a complete interventional spectrum ranging from microscopic inhibition to macroscopic regulation. Ultimately, they aim to achieve effective control of renal IRI as a complex systemic disease.

**Table 1:** Clinical development status of therapeutic strategies targeting the PCD network in renal IRI.

Therapeutic strategy	Target pathway	Agent(s)	Clinical status
Node-specific inhibitors			
Caspase-1 inhibitor	Pyroptosis	VX-765	Phase II (other indications)
Broad-spectrum caspase inhibitor	Pyroptosis	Z-VAD-FMK	Preclinical only
NLRP3 inhibitor	Pyroptosis	MCC950, CY-09	Phase II (MCC950 halted)
GSDMD inhibitor	Pyroptosis	Disulfiram	FDA-approved (alcohol use disorder)
Ferroptosis inhibitor	Ferroptosis	Ferrostatin-1, Liproxstatin-1	Preclinical only
Iron chelator	Ferroptosis	Deferoxamine (DFO)	FDA-approved (iron overload)
ACSL4 inhibitor	Ferroptosis	AS	Preclinical only
RIPK1 inhibitor	Necroptosis	Nec-1f, GSK’872	Preclinical only
RIPK3 inhibitor	Necroptosis	AZD5423	Preclinical only
MLKL inhibitor	Necroptosis	Necrosulfonamide (NSA)	Preclinical only
Network modulation			
Nrf2 activator	Multi-target	Bardoxolone methyl (CDDO-Me)	Phase III (CKD, halted)
Nrf2 activator	Multi-target	Sulforaphane	Clinical (dietary supplement)
Combination therapy			
Ferroptosis + necroptosis inhibition	Dual-target	16-86 + Nec-1s	Preclinical only
Dual inhibitor	Ferroptosis + necroptosis	Zharp1-163	Preclinical only
Upstream modulators			
Mitochondria-targeted antioxidant	Apoptosis	MitoQ	Clinical trials (other)
ER stress inhibitor	Apoptosis/ferroptosis	4-PBA, TUDCA	FDA-approved (4-PBA for urea cycle disorders)
Repurposed clinical drugs			
α2-AR agonist	Ferroptosis	Dexmedetomidine	FDA-approved (sedation)
Anticonvulsant	Necroptosis	Phenytoin	FDA-approved (epilepsy)
Vitamin D analog	Pyroptosis	Calcifediol	FDA-approved (vitamin D deficiency)
Antimalarial	Pyroptosis	Artesunate	FDA-approved (malaria)
COMT inhibitor	Ferroptosis	Entacapone	FDA-approved (Parkinson’s disease)

### 4.1 Node-specific inhibitors: success and the challenge of compensation

It is important to emphasize that most therapeutic strategies discussed in this section are still at the preclinical stage. Most evidence comes from rodent models of renal IRI. And none of these strategies have been approved specifically for this indication. Therefore, while the results from animal models are promising, considerable caution is warranted when extrapolating these findings to human patients.

Various inhibitors have demonstrated efficacy in preclinical models of renal IRI. Inhibitors targeting key nodes of pyroptosis primarily include caspase inhibitors, NLRP3 inflammasome inhibitors and GSDMD inhibitors. VX-765 specifically inhibits caspase-1. It effectively blocks caspase-1-dependent GSDMD activation. This significantly attenuates pyroptosis and the associated inflammatory responses. Ultimately, it ameliorates renal fibrosis (147). The broad-spectrum caspase inhibitor Z-VAD-FMK mitigates tissue injury through multi-target inhibition of pyroptosis (148). MCC950 inhibits the NLRP3 signaling pathway by blocking ASC oligomerization, thereby reducing renal damage and cytokine release (149). CY-09 exerts a renoprotective effect by directly binding to NLRP3 and inhibiting its ATPase activity (150). Disulfiram targets the downstream executioner protein GSDMD. It inhibits pore formation by covalently modifying the Cys191 residue of GSDMD. Additionally, disulfiram antagonizes TLR4 and suppresses the caspase-11-GSDMD pathway, thereby inhibiting pyroptosis at multiple levels (151).

Interventions targeting ferroptosis primarily focus on its core regulatory mechanisms. These interventions utilize specific inhibitors and iron chelators. Ferrostatin-1 directly inhibits ferroptosis. In mouse models, it has been shown to significantly improve renal dysfunction and tubular damage (152). Liproxstatin-1 (Lip-1) suppresses ferroptosis by modulating early growth response protein 1 (EGR1). It also reduces macrophage infiltration and inflammatory cytokine release, thereby effectively mitigating renal IRI (153). The iron chelator deferoxamine (DFO) exerts a renoprotective effect by reducing the intracellular iron burden (154). ACSL4 is a key regulator of ferroptosis and has emerged as a potential therapeutic target. Its specific inhibitor, AS, binds to the glutamine 464 site to inhibit enzyme activity, thereby alleviating lipid peroxidation and ferroptosis (155).

Inhibitors targeting key nodes of necroptosis primarily focus on three core regulatory molecules, including RIPK1, RIPK3 and MLKL. These three form a comprehensive intervention system ranging from upstream kinase inhibition to downstream blockade of the executioner protein. Necrostatin-1f (Nec-1f) inhibits RIPK1 activity. It blocks the necroptotic process and significantly improves survival rates in animal models of renal IRI (156). GSK872 is a selective kinase inhibitor that blocks RIPK3-mediated MLKL phosphorylation, thereby attenuating renal fibrosis (157). AZD5423, also a RIPK3 kinase inhibitor, shows promising prospects for clinical translation (158). Necrosulfonamide (NSA) directly acts on the downstream executioner protein MLKL. It inhibits MLKL oligomerization and membrane pore formation, exerting a renoprotective effect (159). The Hsp90 inhibitor C316–1 inhibits necroptosis by disrupting the interaction between Hsp90 and cell division cycle 37 homolog (CDC37), leading to RIPK1 degradation (160).

### 4.2 Promising multi-target and network modulation approaches

Given the complexity and redundancy of the PCD network, therapies that target a single node often have limited efficacy. Consequently, intervention strategies are gradually shifting toward multi-target approaches. These aim to simultaneously modulate several key nodes. Alternatively, macro-level strategies are being designed to reshape the homeostasis of the entire network. Similar to the node-specific inhibitors discussed above, the multi-target and network modulation strategies described in this section are also predominantly at the preclinical stage. The bulk of the evidence is limited to rodent studies of renal IRI. To date, no therapy has received specific approval for this condition. Nevertheless, they represent promising next-generation strategies that may overcome the limitations of single-node inhibition.

#### 4.2.1 Nrf2 activators: strengthening the core defense network

Nrf2 is a master regulator of the cellular antioxidant response. Under homeostatic conditions, Nrf2 binds to KEAP1 and undergoes ubiquitin-mediated degradation (161). Under oxidative stress, Nrf2 dissociates and translocates to the nucleus. There, it activates the expression of several cytoprotective genes, including HO-1, glutamate-cysteine ligase catalytic subunit (GCLC) and GPX4 (162, 163). Consequently, activating the Nrf2 pathway enhances both GSH biosynthesis and GPX4 expression. This fundamentally strengthens the core antioxidant system that defends against ferroptosis. Furthermore, Nrf2 activation can inhibit the assembly and activation of the NLRP3 inflammasome. It also attenuates inflammatory responses, thereby exerting inhibitory effects on pyroptosis (164–166). Nrf2 activators, such as Bardoxolone Methyl (CDDO-Me) and sulforaphane, have demonstrated efficacy in improving renal function in various kidney disease models. The protective effects of these activators are closely associated with upregulation of GPX4 and inhibition of lipid peroxidation (167, 168).

#### 4.2.2 Combination therapy: synergistic blockade of parallel death pathways

Given the presence of escape or compensation mechanisms within the PCD network, targeting two or more key and interconnected death pathways simultaneously may produce synergistic effects. This approach could yield superior therapeutic outcomes compared to any single-agent therapy. Considering that ferroptosis and necroptosis are often co-activated in renal IRI and exhibit crosstalk, combined inhibition shows promising potential. In a mouse model of renal IRI, concurrent use of the ferroptosis inhibitor 16–86 and the necroptosis inhibitor Necrostatin-1s (Nec-1s) resulted in significantly greater reduction of tubular necrosis. It also led to greater improvement in renal function and more effective lowering of inflammatory cytokine levels compared to either agent alone (95). To achieve combined inhibition more precisely and efficiently, researchers are developing single molecules capable of simultaneously targeting key nodes across multiple death pathways. The compound Zharp1–163 is a dual inhibitor that effectively blocks both ferroptosis and necroptosis. In a mouse model of lipopolysaccharide-induced sepsis-associated AKI, treatment with Zharp1–163 significantly reduced biomarkers of ferroptosis, including 4-hydroxynonenal (4-HNE) and prostaglandin-endoperoxide synthase 2 (PTGS2). It also reduced biomarkers of necroptosis, such as phosphorylated MLKL (p-MLKL). Its renoprotective effect surpassed that of single-target inhibitors, providing proof of concept for the future development of such network-targeting drugs (169).

#### 4.2.3 Upstream modulators: targeting shared stress sources

Multiple PCD pathways share common upstream triggers, including mitochondrial dysfunction, ER stress and ROS burst. Therefore, targeting these shared stress sources can simultaneously attenuate the activation signals for multiple downstream death pathways at the upstream level.

Mitochondria are the primary site of ROS production. They also serve as critical regulatory nodes for ferroptosis, apoptosis and pyroptosis. Mitochondria-targeted antioxidants, like MitoQ, which is a coenzyme Q10 derivative covalently linked to a mitochondrial-targeting sequence, can accumulate within mitochondria and scavenge ROS. Studies have shown that MitoQ pretreatment significantly alleviates apoptosis and oxidative damage in renal IRI. It preserves mitochondrial function, reduces cytochrome c release and inhibits caspase-3 activation (170, 171).

ER stress drives the activation of the UPR pathway and the upregulation of CHOP expression. CHOP can concurrently induce both apoptosis and ferroptosis. Chemical chaperones, like 4-phenylbutyric acid (4-PBA) and tauroursodeoxycholic acid (TUDCA), can alleviate ER stress and restore protein folding homeostasis. In renal IRI models, treatment with 4-PBA or TUDCA has been shown to suppress CHOP expression, attenuate apoptosis and reduce dysfunction in renal tubular epithelial cells (172, 173). This suggests that mitigating the upstream event of ER stress may indirectly inhibit several downstream cell death pathways.

By integrating the multi-faceted strategies outlined above, from activating endogenous defense networks, to combinatory blockade of parallel effector pathways, to stabilizing the function of key organelles, we have the potential to develop more effective and robust therapies to counteract the complex PCD network in renal IRI.

### 4.3 Emerging biotherapies and agents: systematically modulating the death network

Beyond existing therapeutic strategies, a range of natural products, clinical drugs and emerging biotherapies have demonstrated the potential to modulate the cell death network in renal IRI. They target upstream regulatory nodes or exert multi-target effects. Among these, several agents are already approved for non-AKI indications. Examples include dexmedetomidine for sedation, phenytoin for epilepsy and calcifediol for vitamin D deficiency. Other agents such as artesunate, honokiol and brazilin remain at the preclinical stage. Their repurposing potential for renal IRI requires further validation. These diverse interventions, acting on various nodes of the cell death network, provide novel perspectives for kidney protection.

Regarding the pyroptosis pathway, artesunate can inhibit the NLRP3 inflammasome and reduce the expression of pyroptosis-related proteins, including caspase-1 and GSDMD (174). Similarly, calcifediol (25-hydroxyvitamin D3) exerts protective effects by reducing ROS production, inhibiting NF-κB activation and suppressing GSDMD-mediated pyroptosis (175). Furthermore, emerging cell therapies, such as fibroblastic reticular cell-derived exosomes (FRC-Exos), can promote PTEN-induced putative kinase 1 (PINK1)-Parkin-mediated mitophagy. This inhibits NLRP3 inflammasome activation and subsequently suppresses pyroptosis (176). In targeting ferroptosis, the clinically used drug dexmedetomidine is an α2-adrenergic receptor (α2-AR) agonist. It alleviates ferroptosis and associated inflammatory responses by inhibiting ACSL4 (177). Among emerging therapies based on extracellular vesicles, exosomes derived from human urine-derived stem cells (USC-Exo) carry the long non-coding RNA (lncRNA) TUG1. TUG1 regulates ACSL4 expression by interacting with serine/arginine-rich splicing factor 1 (SRSF1), thereby inhibiting ferroptosis (178). Meanwhile, the natural product cyanidin-3-glucoside (C3G) effectively mitigates Erastin-induced ferroptosis through antioxidant mechanisms. Its protective effect is comparable to that of the ferroptosis inhibitor liproxstatin-1 (179). The clinical drug entacapone upregulates SLC7A11 expression by activating the P62-KEAP1-NRF2 pathway, enhancing cellular antioxidant capacity and inhibiting ferroptosis (180). Additionally, some agents also modulate necroptosis. The anticonvulsant drug phenytoin sodium exerts a protective effect by attenuating the kinase activity of RIPK1 and inhibiting necroptosis (181).

These diverse therapeutic approaches exert synergistic inhibitory effects on multiple cell death pathways, including pyroptosis, ferroptosis and necroptosis. They achieve this by modulating upstream hubs, such as oxidative stress and key signaling pathways, or by utilizing agents and biological carriers with multi-target properties. This provides a broader repertoire of pharmacological options and combinatorial treatment strategies for the prevention and management of renal IRI.

## 5 Challenges and future perspectives: towards network pharmacology

**Figure 4:**
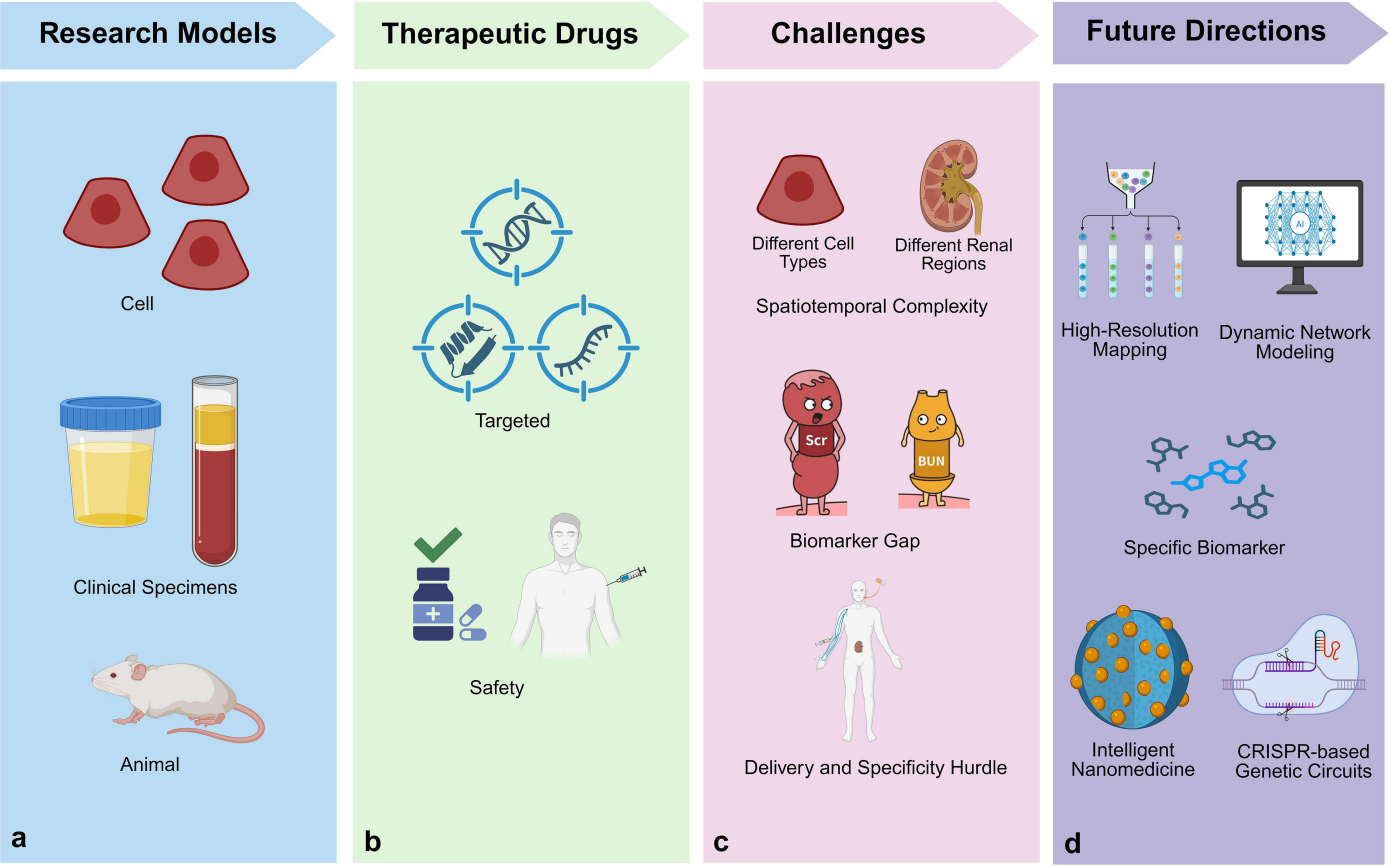
A translational roadmap for targeting the PCD network in renal IRI. **(a)** Multidimensional research models. The foundation relies on a triad of experimental systems. These include *in vitro* cell models to dissect molecular mechanisms, clinical patient samples to validate human relevance and discover biomarkers. They also include *in vivo* animal models to evaluate holistic efficacy and systemic safety. **(b)** Dual core considerations for therapeutic drugs. Successful translation hinges on balancing target specificity with drug safety. Target specificity means precise action on key nodal proteins of the PCD network, such as GPX4 for ferroptosis and GSDMD for pyroptosis. Drug safety involves minimizing off-target effects and long-term toxicity. Both are paramount for clinical viability. **(c)** Three fundamental translational challenges. Spatiotemporal complexity arises from heterogeneity in cell death pathways due to differences in cell types, kidney regions and injury phases. This leads to targeting ambiguity. And there is a biomarker gap. The lack of specific biomarkers that can distinguish the dominant death pathways in real time and non-invasively prevents treatment personalization. There is also a delivery and specificity hurdle. Systemic drug administration causes significant off-target side effects, while kidney-specific and cell-specific targeted delivery systems remain underdeveloped. **(d)** Five frontier research directions and core technologies. High-resolution mapping relies on spatial and single-cell technologies to decipher heterogeneity by charting “death maps” at cellular resolution. Dynamic network modeling uses computational biology to predict key “master switch” nodes within the network. Biomarker-driven clinical trials employ liquid biopsy to enable patient stratification and therapy efficacy monitoring. Intelligent nanomedicine uses stimuli-responsive delivery to achieve spatiotemporally precise drug release at the lesion site. Genetic circuit intervention applies CRISPR regulation to offer the potential for cell-specific “fine-tuning” therapies.

An in-depth understanding of the PCD network has provided a new paradigm for the treatment of renal IRI. However, translating this systemic knowledge into clinically effective interventions still requires bridging the vast gap between basic research and clinical application (**Figure 4**). This translation process faces three core challenges. These include targeting ambiguity due to disease heterogeneity, the lack of dynamic biomarkers to guide precision therapy and the inability of current delivery technologies to meet the need for cell-level precision intervention. These interconnected challenges collectively constitute the main bottleneck in current therapeutic development. Overcoming these bottlenecks demands that we move beyond traditional thinking. It also demands that we develop a new generation of research methods and technologies. This includes utilizing cutting-edge spatial omics technologies to map “death landscapes” at the cellular level, developing specific biomarkers that dynamically reflect the activity of death pathways *in vivo* and designing intelligent delivery systems for spatiotemporally precise drug release. Ultimately, these efforts will converge toward a common goal, namely constructing precision treatment strategies based on individualized “cell death network atlases” for patients. This heralds the true era of network pharmacology.

### 5.1 Challenges

Despite significant advances in understanding PCD networks in renal IRI, several critical challenges must be overcome before these findings can be translated into clinical practice. These challenges span from technical limitations in current experimental approaches to fundamental gaps in our knowledge of network dynamics. They also include the practical hurdles of drug delivery and biomarker development.

Spatiotemporal Complexity. The predominance of specific cell death pathways exhibits high heterogeneity across spatial and temporal dimensions. This dominance varies by cell type. Renal tubular epithelial cells are more susceptible to ferroptosis, whereas pyroptosis may predominate in macrophages (182). It also differs by renal region. The high metabolic demand and hypoxic environment of the medulla may render it more sensitive to certain death modalities (183). Furthermore, it changes with the phase of injury. The ischemic phase may be characterized primarily by apoptosis and energy metabolism collapse, while the reperfusion phase may trigger intense inflammatory and oxidative death (184). Current bulk tissue analysis techniques obscure this critical heterogeneity. This makes it difficult to identify the key cell populations driving injury and the precise molecular events involved.

Biomarker Gap. We lack sensitive and specific biomarkers to map the active cell death pathways in patients in real time. Conventional renal function markers, like Scr and BUN, merely indicate functional impairment. They cannot distinguish whether the injury is predominantly driven by pyroptosis, ferroptosis, necroptosis or other pathways. For instance, there are currently no validated biomarkers to specifically detect pyroptosis, ferroptosis or necroptosis in patients with renal IRI. Examples of such biomarkers would include plasma GSDMD-N for pyroptosis, specific oxidized phospholipids for ferroptosis and p-MLKL for necroptosis. This confines treatment decisions to a “one-size-fits-all” paradigm. It prevents personalized medication based on an individual patien’s real-time “cell death pathway profile”. Consequently, the success rate of targeted therapies is significantly limited (185).

Delivery and Specificity. Even if effective targets and drugs are identified, achieving kidney-specific or even cell type-specific drug delivery remains an unsolved challenge. Systemic administration often leads to unacceptable side effects due to off-target effects. Furthermore, the kidney’s complex structure and hemodynamic characteristics make targeted drug enrichment difficult. Developing safe and efficient targeted delivery systems is therefore an essential pathway to avoid systemic toxicity and improve the therapeutic index (186).

### 5.2 Future directions

High-Resolution Mapping. Future research should systematically apply spatial transcriptomics, single-cell multi-omics and multiplexed immunofluorescence imaging to renal IRI tissues from both humans and animal models. The goal is to construct a cell-resolution activity map of the PCD network across both temporal and spatial dimensions. This will allow precise identification of “hotspot regions” where specific death pathways are active and key vulnerable cell subpopulations. Thus, it will provide a “battle map” for precise intervention (187).

Dynamic Network Modeling. Based on high-throughput mapping data, dynamic computational models of the PCD network in renal IRI should be constructed. These models will simulate the interactions and compensatory relationships between different death pathways. Through network perturbation analysis, they can predict key control nodes, also called “master switches”, that exert a global influence on the entire network. These nodes may not be the terminal executioner proteins of a single death pathway. Insteda, they may be common upstream signals, such as specific redox sensors or metabolites, that regulate multiple pathways. Targeting such nodes might reshape the entire network more efficiently than inhibiting a single pathway (188).

Biomarker-Driven Clinical Trials. Future efforts must focus on developing non-invasive and dynamic detection methods capable of reflecting the dominant PCD pathways *in vivo*. For example, cleaved GSDMD-N serves as a pyroptosis marker. Its plasma levels could identify patients with active inflammasome signaling. Specific oxidized phospholipid species, such as PE-AA-OH, which is a downstream product of ferroptosis, could serve as a pharmacodynamic readout of lipid peroxidation. In addition, p-MLKL acts as a necroptosis executioner and could be a candidate plasma marker. Beyond blood-based markers, liquid biopsy approaches using urinary exosomes represent a particularly attractive non-invasive tool for renal IRI (189). Urinary exosomes can carry cargo derived from injured renal tubular epithelial cells, including GSDMD-N, p-MLKL, GPX4 and lipid peroxidation products. Monitoring these exosomal cargoes in serial urine samples could enable real-time, kidney-specific assessment of the dominant death pathway activity, without the need for invasive renal biopsy. By utilizing such biomarkers, patients with AKI could be precisely stratified in clinical trials. This work paves the way for targeted therapeutic trials. This approach would significantly enhance the success rate of clinical trials and the response rate to investigational drugs (190).

Intelligent Nanomedicine. Design stimulus-responsive nanoparticles as drug carriers. These particles can achieve preferential accumulation in the injured kidney through the size effect or via surface-modified targeting ligands. More importantly, they can sense the local pathological microenvironment, including low pH, high ROS or specific enzyme activity. They can use these cues as triggers to release drug combinations in a sequential or synergistic manner, in a controlled fashion, at the right time and location. This enables “on-demand therapy”, maximizing efficacy while minimizing side effects (191). 

Genetic Circuit Intervention. Explore the use of CRISPR-based gene regulation technologies, which offer unprecedented potential for spatiotemporal precision in intervention. By designing specific guide RNAs and regulatory modules, the expression of key network nodes, such as GPX4 and GSDME, can be dynamically fine-tuned in a time- and cell-specific manner during the injury process. This “fine-tuning” of critical network nodes, rather than a simple “on/off” switch, may better align with the logic of physiological repair. It paves the way for truly “cell-precision medicine” (192).

Potential Risks and Ethical Considerations. While the future directions outlined above hold great promise, they also present potential risks that warrant consideration. High-resolution single-cell and spatial omics technologies generate vast amounts of data. This raises important questions about data management, interpretation and patient privacy when applied to human samples. The development of intelligent nanomedicines and genetic circuit interventions, while innovative, may introduce unforeseen off-target effects or long-term safety concerns. For instance, CRISPR-based gene regulation could potentially lead to permanent genomic alterations if not carefully controlled. Moreover, the translation of network-based combination therapies into clinical practice will require rigorous safety evaluations. This is needed to avoid unintended synergies that might exacerbate tissue injury rather than mitigate it. Therefore, future research should not only focus on technological innovation but also prioritize the development of robust safety assessment frameworks and ethical guidelines for these emerging interventions.

## 6 Conclusion

The pathogenesis of renal IRI is best understood as the coordinated output of a dynamic, interconnected PCD network. It is not a struggle among isolated death pathways. Pyroptosis, ferroptosis, cuproptosis and necroptosis are interconnected through shared triggers, molecular switches and positive feedback loops. Together, they form a system that amplifies injury. While targeting individual network nodes has shown promise, therapeutic breakthroughs will likely require strategies grounded in a network-level understanding. They include rational combination therapies, multi-target agents and ultimately interventions that are precise in both space and time, guided by high-resolution patient data. Embracing this network paradigm is crucial for translating our complex mechanistic understanding into effective treatments for renal IRI and beyond.

## Glossary

AKI: Acute kidney injury
ASC: Apoptosis-associated speck-like protein containing a CARD
AIM2: Melanoma 2
ACSL4: Acyl-CoA synthetase long-chain-fatty-acid--CoA ligase 4
AMPK: Protein kinase AMP-activated catalytic subunit alpha 1
ATF4: Activating transcription factor 4
ATG12: Autophagy-related protein 12
ATG5: Autophagy protein 5
ATG3: Autophagy-related protein 3
BUN: Blood urea nitrogen
Bcl-2: B-cell lymphoma 2
BAX: BCL2 associated X protein
Bcl-xL: B-cell lymphoma extra-large
CKD: Chronic kidney disease
c-FLIP: cellular FLICE-like inhibitory protein
CHOP: DNA damage inducible transcript 3
CaMKII: Calcium/calmodulin-dependent protein kinase
CYLD: Cylindromatosis
CDC37: Cell division cycle 37 homolog
C3G: Cyanidin-3-glucoside
DAMPs: Damage-associated molecular patterns
DISC: Death-inducing signaling complex
DLAT: dihydrolipoamide S-acetyltransferase
DPP4: Dipeptidyl peptidase IV
Drp1: Density-regulated protein
DKD: Diabetic kidney disease
DR5: Tumor necrosis factor receptor superfamily member 10B
DFO: Deferoxamine
ER-phagy: Reticulophagy
ER: Endoplasmic reticulum
EMT: Epithelial-mesenchymal transition
EGR1: Early growth response protein 1
FADD: FAS-associated death domain protein
Fap-1: Fas-associated protein-tyrosine phosphatase 1
Fe-S: Iron-sulfur
FOS: Protein c-Fos
FRC-Exos: Fibroblastic reticular cell-derived exosomes
GSDMD: Gasdermin-D
GSDME: Gasdermin-E
GSH: Glutathione
GPX4: Glutathione peroxidase 4
GCN2: eIF-2-alpha kinase
HMGB1: High mobility group protein B1
HO-1: Heme oxygenase-1
IRI: Ischemia-reperfusion injury
IRGM: Immunity-related GTPase family M protein
ISCU: Iron-sulfur cluster assembly enzyme
KEAP1: Kelch-like ECH-associated protein 1
LGALS9: Galectin-9
Lip-1: Liproxstatin-1
LncRNA: Long non-coding RNA
MLKL: Mixed lineage kinase domain-like protein
mtROS: Mitochondrial ROS
mTOR: Mammalian target of rapamycin
MPTP: Mitochondrial permeability transition pore
Mcl-1: Myeloid cell leukemia 1
MOMP: Mitochondrial outer membrane permeabilization
NLRP3: NLR family pyrin domain containing 3
NF-κB: Nuclear factor kappa-B
Nrf2: Nuclear factor erythroid 2-related factor 2
NFU1: HIRA-interacting protein 5
Nec-1f: Necrostatin-1f
NSA: Necrosulfonamide
Nec-1s: Necrostatin-1s
PCD: Programmed cell death
P62: Sequestosome 1
PUMA: P53-upregulated modulator of apoptosis
P53: Cellular tumor antigen p53
PGAM5: Phosphoglycerate mutase family member 5
PTGS2: Prostaglandin-endoperoxide synthase 2
PINK1: PTEN-induced putative kinase 1
P-MLKL: Phosphorylated MLKL
PTGS2: Prostaglandin G/H synthase 2
RIPK1: Receptor-interacting serine/threonine-protein kinase 1
RIPK3: Receptor-interacting serine/threonine-protein kinase 3
ROS: Reactive oxygen species
RAGE: Advanced glycosylation end products
Scr: Serum creatinine
STAT3: Signal transducer and activator of transcription 3
SRSF1: Serine/arginine-rich splicing factor 1
TCA: Tricarboxylic acid
TLR4: Toll-like receptor 4
TRIM20: Tripartite motif containing 20
TWEAK: TNF-related weak inducer of apoptosis
TNF: Tumor necrosis factor
TUDCA: Tauroursodeoxycholic acid
TRAIL: Tumor necrosis factor ligand superfamily member 10
USP18: Ubiquitin specific peptidase 18
USP5: Ubiquitin specific peptidase 5
UPR: Unfolded protein response
ULK1: Unc-51-like kinase 1
USC-Exo: Exosomes derived from human urine-derived stem cells
4-HNE: 4-hydroxynonenal
4-PBA: 4-phenylbutyric acid
α2-AR: α2-adrenergic receptor

## References

[1] Sewell WH Koth DR Huggins CE . Ventricular fibrillation in dogs after sudden return of flow to the coronary artery. Surgery. (1955) 38:1050–3. 13274263

[2] Eltzschig HK Eckle T . Ischemia and reperfusion--from mechanism to translation. Nat Med. (2011) 17:1391–401. doi: 10.1038/nm.2507 22064429 PMC3886192

[3] Menke J Sollinger D Schamberger B Heemann U Lutz J . The effect of ischemia/reperfusion on the kidney graft. Curr Opin Organ Transplant. (2014) 19:395–400. doi: 10.1097/mot.0000000000000090 24905021

[4] Lafrance JP Miller DR . Acute kidney injury associates with increased long-term mortality. J Am Soc Nephrol. (2010) 21:345–52. doi: 10.1681/ASN.2009060636 20019168 PMC2834549

[5] Malek M Nematbakhsh M . Renal ischemia/reperfusion injury; from pathophysiology to treatment. J Renal Inj Prev. (2015) 4:20–7. doi: 10.12861/jrip.2015.06 26060833 PMC4459724

[6] Kroemer G Galluzzi L Vandenabeele P Abrams J Alnemri ES Baehrecke EH . Classification of cell death: recommendations of the Nomenclature Committee on Cell Death 2009. Cell Death Differ. (2009) 16:3–11. doi: 10.1038/cdd.2008.150 18846107 PMC2744427

[7] Yuan J Ofengeim D . A guide to cell death pathways. Nat Rev Mol Cell Biol. (2024) 25:379–95. doi: 10.1038/s41580-023-00689-6 38110635

[8] Tang D Kang R Berghe V Vandenabeele P Kroemer G . The molecular machinery of regulated cell death. Cell Res. (2019) 29:347–64. doi: 10.1038/s41422-019-0164-5 30948788 PMC6796845

[9] Galluzzi L Vitale I Aaronson SA Abrams JM Adam D Agostinis P . Molecular mechanisms of cell death: recommendations of the Nomenclature Committee on Cell Death 2018. Cell Death Differ. (2018) 25:486–541. doi: 10.1038/s41418-017-0012-4 29362479 PMC5864239

[10] Li C Yu Y Zhu S Hu Y Ling X Xu L . The emerging role of regulated cell death in ischemia and reperfusion-induced acute kidney injury: current evidence and future perspectives. Cell Death Discov. (2024) 10:216. doi: 10.1038/s41420-024-01979-4 38704372 PMC11069531

[11] Ying Y Padanilam BJ . Regulation of necrotic cell death: p53, PARP1 and cyclophilin D-overlapping pathways of regulated necrosis? Cell Mol Life Sci. (2016) 73:2309–24. doi: 10.1007/s00018-016-2202-5 27048819 PMC5490387

[12] Shi J Zhao Y Wang K Shi X Wang Y Huang H . Cleavage of GSDMD by inflammatory caspases determines pyroptotic cell death. Nature. (2015) 526:660–5. doi: 10.1038/nature15514 26375003

[13] Ning J Wang J Sun X Li H Cheng F . TRIM44 alleviates renal ischemia-reperfusion injury by inhibiting pyroptosis through the NLRP3 pathway. Mol Immunol. (2025) 178:20–31. doi: 10.1016/j.molimm.2025.01.003 39813853

[14] Chen L Fang H Li X Yu P Guan Y Xiao C . Connexin32 gap junction channels deliver miR155-3p to mediate pyroptosis in renal ischemia-reperfusion injury. Cell Commun Signal. (2024) 22:121. doi: 10.1186/s12964-023-01443-3 38347637 PMC10863161

[15] Yang JR Yao FH Zhang JG Ji ZY Li KL Zhan J . Ischemia-reperfusion induces renal tubule pyroptosis via the CHOP-caspase-11 pathway. Am J Physiol Renal Physiol. (2014) 306:F75–84. doi: 10.1152/ajprenal.00117.2013 24133119

[16] Ma N Lu H Li N Ni W Zhang W Liu Q . CHOP-mediated Gasdermin E expression promotes pyroptosis, inflammation, and mitochondrial damage in renal ischemia-reperfusion injury. Cell Death Dis. (2024) 15:163. doi: 10.21203/rs.3.rs-3380049/v1 38388468 PMC10883957

[17] Li J Cao F Yin HL Huang ZJ Lin ZT Mao N . Ferroptosis: past, present and future. Cell Death Dis. (2020) 11:88. doi: 10.1038/s41419-020-2298-2 32015325 PMC6997353

[18] Yang WS SriRamaratnam R Welsch ME Shimada K Skouta R Viswanathan VS . Regulation of ferroptotic cancer cell death by GPX4. Cell. (2014) 156:317–31. doi: 10.1016/j.cell.2013.12.010 24439385 PMC4076414

[19] Yang WS Stockwell BR . Ferroptosis: Death by Lipid Peroxidation. Trends Cell Biol. (2016) 26:165–76. doi: 10.1016/j.tcb.2015.10.014 26653790 PMC4764384

[20] Chen X Li J Kang R Klionsky DJ Tang D . Ferroptosis: machinery and regulation. Autophagy. (2021) 17:2054–81. doi: 10.1080/15548627.2020.1810918 32804006 PMC8496712

[21] Thapa K Singh TG Kaur A . Targeting ferroptosis in ischemia/reperfusion renal injury. Naunyn Schmiedebergs Arch Pharmacol. (2022) 395:1331–41. doi: 10.1007/s00210-022-02277-5 35920897

[22] Wang W Chen J Zhan L Zou H Wang L Guo M . Iron and ferroptosis in kidney disease: molecular and metabolic mechanisms. Front Immunol. (2025) 16:1531577. doi: 10.3389/fimmu.2025.1531577 39975561 PMC11835690

[23] Qi Y Zheng J Zi Y Song W Chen X Cao S . Loureirin C improves mitochondrial function by promoting NRF2 nuclear translocation to attenuate oxidative damage caused by renal ischemia-reperfusion injury. Int Immunopharmacol. (2024) 138:112596. doi: 10.1016/j.intimp.2024.112596 38981224

[24] Zhao Z Li G Wang Y Li Y Xu H Liu W . Cytoplasmic HMGB1 induces renal tubular ferroptosis after ischemia/reperfusion. Int Immunopharmacol. (2023) 116:109757. doi: 10.1016/j.intimp.2023.109757 36731154

[25] Tsvetkov P Coy S Petrova B Dreishpoon M Verma A Abdusamad M . Copper induces cell death by targeting lipoylated TCA cycle proteins. Science. (2022) 375:1254–61. doi: 10.1126/science.abf0529 35298263 PMC9273333

[26] Saifi MA Godugu C . Copper chelation therapy inhibits renal fibrosis by modulating copper transport proteins. Biofactors. (2022) 48:934–45. doi: 10.1002/biof.1837 35322483

[27] Chen S Chen T Xu C Yu X Shi J Yang C . Iron overload exaggerates renal ischemia-reperfusion injury by promoting tubular cuproptosis via interrupting function of LIAS. Redox Biol. (2025) 86:103795. doi: 10.1016/j.redox.2025.103795 40753758 PMC12375214

[28] Ray CA Pickup DJ . The mode of death of pig kidney cells infected with cowpox virus is governed by the expression of the crmA gene. Virology. (1996) 217:384–91. doi: 10.1006/viro.1996.0128 8599227

[29] Teng X Degterev A Jagtap P Xing X Choi S Denu R . Structure-activity relationship study of novel necroptosis inhibitors. Bioorg Med Chem Lett. (2005) 15:5039–44. doi: 10.1016/j.bmcl.2005.07.077 16153840

[30] Linkermann A Bräsen JH Himmerkus N Liu S Huber TB Kunzendorf U . Rip1 (receptor-interacting protein kinase 1) mediates necroptosis and contributes to renal ischemia/reperfusion injury. Kidney Int. (2012) 81:751–61. doi: 10.1038/ki.2011.450 22237751

[31] Liu W Chen B Wang Y Meng C Huang H Huang XR . RGMb protects against acute kidney injury by inhibiting tubular cell necroptosis via an MLKL-dependent mechanism. Proc Natl Acad Sci USA. (2018) 115:E1475–84. doi: 10.1073/pnas.1716959115 29382757 PMC5816182

[32] Fu ZJ Wang ZY Xu L Chen XH Li XX Liao WT . HIF-1α-BNIP3-mediated mitophagy in tubular cells protects against renal ischemia/reperfusion injury. Redox Biol. (2020) 36:101671. doi: 10.1016/j.redox.2020.101671 32829253 PMC7452120

[33] Decuypere JP Ceulemans LJ Agostinis P Monbaliu D Naesens M Pirenne J . Autophagy and the Kidney: Implications for Ischemia-Reperfusion Injury and Therapy. Am J Kidney Dis. (2015) 66:699–709. doi: 10.1053/j.ajkd.2015.05.021 26169721

[34] Kang S Fernandes-Alnemri T Rogers C Mayes L Wang Y Dillon C . Caspase-8 scaffolding function and MLKL regulate NLRP3 inflammasome activation downstream of TLR3. Nat Commun. (2015) 6:7515. doi: 10.1038/ncomms8515 26104484 PMC4480782

[35] Gutierrez KD Davis MA Daniels BP Olsen TM Ralli-Jain P Tait SW . MLKL Activation Triggers NLRP3-Mediated Processing and Release of IL-1β Independently of Gasdermin-D. J Immunol. (2017) 198:2156–64. doi: 10.4049/jimmunol.1601757 28130493 PMC5321867

[36] Tonnus W Maremonti F Belavgeni A Latk M Kusunoki Y Brucker A . Gasdermin D-deficient mice are hypersensitive to acute kidney injury. Cell Death Dis. (2022) 13:792. doi: 10.1038/s41419-022-05230-9 36109515 PMC9478139

[37] Huang Q Shi Z Zheng D Chen H Huang Q . Astragalin Inhibits Oxidative Stress-Induced Pyroptosis and Apoptosis in Mouse Models of Renal Ischemia/Reperfusion Injury by Activating the SIRT1/Nrf2 Pathway. Phytother Res. (2025) 39:5025–42. doi: 10.1002/ptr.8527 40902986

[38] Zhang WH Wang X Narayanan M Zhang Y Huo C Reed JC . Fundamental role of the Rip2/caspase-1 pathway in hypoxia and ischemia-induced neuronal cell death. Proc Natl Acad Sci USA. (2003) 100:16012–7. doi: 10.1073/pnas.2534856100 14663141 PMC307684

[39] Taabazuing CY Okondo MC Bachovchin DA . Pyroptosis and Apoptosis Pathways Engage in Bidirectional Crosstalk in Monocytes and Macrophages. Cell Chem Biol. (2017) 24:507–514.e4. doi: 10.1016/j.chembiol.2017.03.009 28392147 PMC5467448

[40] Kang SJ Wang S Hara H Peterson EP Namura S Amin-Hanjani S . Dual role of caspase-11 in mediating activation of caspase-1 and caspase-3 under pathological conditions. J Cell Biol. (2000) 149:613–22. doi: 10.1083/jcb.149.3.613 10791975 PMC2174843

[41] Zhang W Zhu C Liao Y Zhou M Xu W Zou Z . Caspase-8 in inflammatory diseases: a potential therapeutic target. Cell Mol Biol Lett. (2024) 29:130. doi: 10.1186/s11658-024-00646-x 39379817 PMC11463096

[42] Wu YH Kuo WC Wu YJ Yang KT Chen ST Jiang ST . Participation of c-FLIP in NLRP3 and AIM2 inflammasome activation. Cell Death Differ. (2014) 21:451–61. doi: 10.4049/jimmunol.192.supp.125.2 PMC3921593 24270411

[43] Gurung P Anand PK Malireddi RK Vande Walle L Van Opdenbosch N Dillon CP . FADD and caspase-8 mediate priming and activation of the canonical and noncanonical Nlrp3 inflammasomes. J Immunol. (2014) 192:1835–46. doi: 10.4049/jimmunol.192.supp.120.1 PMC3933570 24453255

[44] Jiang M Qi L Li L Li Y . The caspase-3/GSDME signal pathway as a switch between apoptosis and pyroptosis in cancer. Cell Death Discov. (2020) 6:112. doi: 10.1038/s41420-020-00349-0 33133646 PMC7595122

[45] Chen KW Demarco B Heilig R Shkarina K Boettcher A Farady CJ . Extrinsic and intrinsic apoptosis activate pannexin-1 to drive NLRP3 inflammasome assembly. EMBO J. (2019) 38:e101638. doi: 10.15252/embj.2019101638 30902848 PMC6517827

[46] Elrashidy RA Mohamad HE Aal SMA Mohamed SR Tolba SM Mahmoud YK . Repurposing Secukinumab and Dapagliflozin as Candidate Therapies to Mitigate the Renal Toxicity of Sunitinib in Rats Through Suppressing IL-17-Mediated Pyroptosis and Promoting Autophagy. J Biochem Mol Toxicol. (2025) 39:e70204. doi: 10.1002/jbt.70204 40059817

[47] Pang Q Wang P Pan Y Dong X Zhou T Song X . Irisin protects against vascular calcification by activating autophagy and inhibiting NLRP3-mediated vascular smooth muscle cell pyroptosis in chronic kidney disease. Cell Death Dis. (2022) 13:283. doi: 10.1038/s41419-022-04735-7 35354793 PMC8967887

[48] Hu Y Wang K Xu J Wan G Zhao Y Chen Y . mTOR-Mediated Autophagy Regulates Cadmium-Induced Kidney Injury via Pyroptosis. Int J Mol Sci. (2025) 26:2589. doi: 10.3390/ijms26062589 40141229 PMC11942160

[49] Zhu X Li S Lin Q Shao X Wu J Zhang W . αKlotho protein has therapeutic activity in contrast-induced acute kidney injury by limiting NLRP3 inflammasome-mediated pyroptosis and promoting autophagy. Pharmacol Res. (2021) 167:105531. doi: 10.1016/j.phrs.2021.105531 33675964

[50] Li MY Zhu XL Zhao BX Shi L Wang W Hu W . Adrenomedullin alleviates the pyroptosis of Leydig cells by promoting autophagy via the ROS-AMPK-mTOR axis. Cell Death Dis. (2019) 10:489. doi: 10.1038/s41419-019-1728-5 31222000 PMC6586845

[51] Zhang D Zhang Y Pan J Cao J Sun X Li X . Degradation of NLRP3 by p62-dependent-autophagy improves cognitive function in Alzheimer's disease by maintaining the phagocytic function of microglia. CNS Neurosci Ther. (2023) 29:2826–42. doi: 10.1111/cns.14219 37072933 PMC10493665

[52] Kimura T Jain A Choi SW Mandell MA Johansen T Deretic V . TRIM-directed selective autophagy regulates immune activation. Autophagy. (2017) 13:989–90. doi: 10.1080/15548627.2016.1154254 26983397 PMC5446080

[53] Wang L Li M Lian G Yang S Wu Y Cui J . USP18 antagonizes pyroptosis by facilitating selective autophagic degradation of Gasdermin D. Res (Wash D C). (2024) 7:380. doi: 10.34133/research.0380 38779488 PMC11109516

[54] Mehto S Jena KK Nath P Chauhan S Kolapalli SP Das SK . The Crohn's disease risk factor IRGM limits NLRP3 inflammasome activation by impeding its assembly and by mediating its selective autophagy. Mol Cell. (2019) 73:429–445.e7. doi: 10.1016/j.molcel.2018.11.018 30612879 PMC6372082

[55] Cai B Zhao J Zhang Y Liu Y Ma C Yi F . USP5 attenuates NLRP3 inflammasome activation by promoting autophagic degradation of NLRP3. Autophagy. (2022) 18:990–1004. doi: 10.1080/15548627.2021.1965426 34486483 PMC9196652

[56] Wang W Qin Y Song H Wang L Jia M Zhao C . Galectin-9 targets NLRP3 for autophagic degradation to limit inflammation. J Immunol. (2021) 206:2692–9. doi: 10.4049/jimmunol.2001404 33963043

[57] Sun C Liu J Li H Yan Y . Melatonin attenuates ischemia-reperfusion-induced acute kidney injury by regulating abnormal autophagy and pyroptosis through SIRT1-mediated p53 deacetylation. Int Immunopharmacol. (2025) 162:115092. doi: 10.1016/j.intimp.2025.115092 40561830

[58] Hu Y Shi Y Chen H Tao M Zhou X Li J . Blockade of autophagy prevents the progression of hyperuricemic nephropathy through inhibiting NLRP3 inflammasome-mediated pyroptosis. Front Immunol. (2022) 13:858494. doi: 10.3389/fimmu.2022.858494 35309342 PMC8924517

[59] Wan F Zhong G Wu S Jiang X Liao J Zhang X . Arsenic and antimony co-induced nephrotoxicity via autophagy and pyroptosis through ROS-mediated pathway *in vivo* and *in vitro*. Ecotoxicol Environ Saf. (2021) 221:112442. doi: 10.1016/j.ecoenv.2021.112442 34166936

[60] Wang L Shao Z Wang N Liu W Zhang L Wang Y . Receptor-interacting protein kinase 1 confers autophagic promotion of gasdermin E-mediated pyroptosis in aristolochic acid-induced acute kidney injury. Ecotoxicol Environ Saf. (2024) 284:116944. doi: 10.1016/j.ecoenv.2024.116944 39208575

[61] Liu W Gan Y Ding Y Zhang L Jiao X Liu L . Autophagy promotes GSDME-mediated pyroptosis via intrinsic and extrinsic apoptotic pathways in cobalt chloride-induced hypoxia reoxygenation-acute kidney injury. Ecotoxicol Environ Saf. (2022) 242:113881. doi: 10.1016/j.ecoenv.2022.113881 35863214

[62] Ding T Zhang P Wang K Du P Duan B . FGF4 alleviates renal injury caused by ischemia-reperfusion(I/R) by inhibiting ferroptosis and pyroptosis. Peptides. (2025) 192:171438. doi: 10.1016/j.peptides.2025.171438 40840789

[63] Liu H Lin H He Y Shi S Ni J Zhao L . Novel epigenetic biomarkers following ferroptosis and pyroptosis in a hypobaric hypoxia-induced renal injury model. Arch Biochem Biophys. (2025) 774:110637. doi: 10.1016/j.abb.2025.110637 41067610

[64] Liu S Xu Y Yao X Cao H Zhou H Luo J . Perillaldehyde ameliorates sepsis-associated acute kidney injury via inhibiting HSP90AA1-mediated ferroptosis and pyroptosis: Molecular structure and protein interaction of HSP90AA1. Int J Biol Macromol. (2025) 304:140954. doi: 10.1016/j.ijbiomac.2025.140954 39947536

[65] Zhang D Wu C Ba D Wang N Wang Y Li X . Ferroptosis contribute to neonicotinoid imidacloprid-evoked pyroptosis by activating the HMGB1-RAGE/TLR4-NF-κB signaling pathway. Ecotoxicol Environ Saf. (2023) 253:114655. doi: 10.1016/j.ecoenv.2023.114655 36812867

[66] Chen K Tang Y Lan L Li M Lu Z . Autophagy mediated FTH1 degradation activates gasdermin E dependent pyroptosis contributing to diquat induced kidney injury. Food Chem Toxicol. (2024) 184:114411. doi: 10.1016/j.fct.2023.114411 38128689

[67] Wang L Yang F Hu M Chen G Wang Y Xue H . GPX4 utilization by selenium is required to alleviate cadmium-induced ferroptosis and pyroptosis in sheep kidney. Environ Toxicol. (2023) 38:962–74. doi: 10.1002/tox.23740 36655595

[68] Battino M Giampieri F Pistollato F Sureda A de Oliveira MR Pittalà V . Nrf2 as regulator of innate immunity: A molecular Swiss army knife! Biotechnol Adv. (2018) 36:358–70. doi: 10.1016/j.biotechadv.2017.12.012 29277308

[69] Fan Z Wirth AK Chen D Wruck CJ Rauh M Buchfelder M . Nrf2-Keap1 pathway promotes cell proliferation and diminishes ferroptosis. Oncogenesis. (2017) 6:e371. doi: 10.1038/oncsis.2017.65 28805788 PMC5608917

[70] Yan Z Qi W Zhan J Lin Z Lin J Xue X . Activating Nrf2 signalling alleviates osteoarthritis development by inhibiting inflammasome activation. J Cell Mol Med. (2020) 24:13046–57. doi: 10.1111/jcmm.15905 32965793 PMC7701566

[71] Hou Y Wang S Jiang L Sun X Li J Wang N . Patulin induces acute kidney injury in mice through autophagy-ferroptosis pathway. J Agric Food Chem. (2022) 70:6213–23. doi: 10.1021/acs.jafc.1c08349 35543324

[72] Liu L Wei Q Wang R Sun H He S Tang L . Rab7-regulated ferroptosis contributes to tubular epithelial cells injury by degradation of GPX4 via chaperone-mediated autophagy in AKI. Am J Physiol Cell Physiol. (2025) 328:C699–709. doi: 10.1152/ajpcell.00636.2023 39932439

[73] Chen C Wang D Yu Y Zhao T Min N Wu Y . Legumain promotes tubular ferroptosis by facilitating chaperone-mediated autophagy of GPX4 in AKI. Cell Death Dis. (2021) 12:65. doi: 10.1038/s41419-020-03362-4 33431801 PMC7801434

[74] Wang L Klionsky DJ Shen HM . The emerging mechanisms and functions of microautophagy. Nat Rev Mol Cell Biol. (2023) 24:186–203. doi: 10.1038/s41580-022-00529-z 36097284

[75] Sui M Xu D Zhao W Lu H Chen R Duan Y . CIRBP promotes ferroptosis by interacting with ELAVL1 and activating ferritinophagy during renal ischaemia-reperfusion injury. J Cell Mol Med. (2021) 25:6203–16. doi: 10.1111/jcmm.16567 34114349 PMC8256344

[76] Lin Q Li S Jin H Cai H Zhu X Yang Y . Mitophagy alleviates cisplatin-induced renal tubular epithelial cell ferroptosis through ROS/HO-1/GPX4 axis. Int J Biol Sci. (2023) 19:1192–210. doi: 10.7150/ijbs.80775 36923942 PMC10008689

[77] Singh R Kaushik S Wang Y Xiang Y Novak I Komatsu M . Autophagy regulates lipid metabolism. Nature. (2009) 458:1131–5. doi: 10.1038/nature07976 19339967 PMC2676208

[78] Bai Y Meng L Han L Jia Y Zhao Y Gao H . Lipid storage and lipophagy regulates ferroptosis. Biochem Biophys Res Commun. (2019) 508:997–1003. doi: 10.1016/j.bbrc.2018.12.039 30545638

[79] Bernales S McDonald KL Walter P . Autophagy counterbalances endoplasmic reticulum expansion during the unfolded protein response. PloS Biol. (2006) 4:e423. doi: 10.1371/journal.pbio.0040423 17132049 PMC1661684

[80] Liu Z Ma C Wang Q Yang H Lu Z Bi T . Targeting FAM134B-mediated reticulophagy activates sorafenib-induced ferroptosis in hepatocellular carcinoma. Biochem Biophys Res Commun. (2022) 589:247–53. doi: 10.1016/j.bbrc.2021.12.019 34929448

[81] Chae CW Yoon JH Lim JR Park JY Cho JH Jung YH . TRIM16-mediated lysophagy suppresses high-glucose-accumulated neuronal Aβ. Autophagy. (2023) 19:2752–68. doi: 10.1080/15548627.2023.2229659 37357416 PMC10472864

[82] Li S Liao Z Yin H Liu O Hua W Wu X . G3BP1 coordinates lysophagy activity to protect against compression-induced cell ferroptosis during intervertebral disc degeneration. Cell Prolif. (2023) 56:e13368. doi: 10.1111/cpr.13368 36450665 PMC9977669

[83] Wang X Chen T Chen S Zhang J Cai L Liu C . STING aggravates ferroptosis-dependent myocardial ischemia-reperfusion injury by targeting GPX4 for autophagic degradation. Signal Transduct Target Ther. (2025) 10:136. doi: 10.1038/s41392-025-02216-9 40274801 PMC12022026

[84] Doll S Proneth B Tyurina YY Panzilius E Kobayashi S Ingold I . ACSL4 dictates ferroptosis sensitivity by shaping cellular lipid composition. Nat Chem Biol. (2017) 13:91–8. doi: 10.1038/nchembio.2239 27842070 PMC5610546

[85] Helfenberger KE Argentino GF Benzo Y Herrera LM Finocchietto P Poderoso C . Angiotensin II regulates mitochondrial mTOR pathway activity dependent on acyl-CoA synthetase 4 in adrenocortical cells. Endocrinology. (2022) 163:bqac170. doi: 10.1210/endocr/bqac170 36256598

[86] Zhang C Li A Gao S Zhang X Xiao H . The TIP30 protein complex, arachidonic acid and coenzyme A are required for vesicle membrane fusion. PloS One. (2011) 6:e21233. doi: 10.1371/journal.pone.0021233 21731680 PMC3123320

[87] Liu C Sun W Zhu T Shi S Zhang J Wang J . Glia maturation factor-β induces ferroptosis by impairing chaperone-mediated autophagic degradation of ACSL4 in early diabetic retinopathy. Redox Biol. (2022) 52:102292. doi: 10.1016/j.redox.2022.102292 35325805 PMC8942824

[88] Chen Z Chen G Shi J Jin L . BMAL1 alleviates sepsis-induced acute kidney injury by inhibiting apoptosis, ferroptosis and inflammation. Hereditas. (2025) 162:208. doi: 10.1186/s41065-025-00583-5 41088456 PMC12522825

[89] Lee YS Lee DH Choudry HA Bartlett DL Lee YJ . Ferroptosis-induced endoplasmic reticulum stress: Cross-talk between ferroptosis and apoptosis. Mol Cancer Res. (2018) 16:1073–6. doi: 10.1158/1541-7786.mcr-18-0055 29592897 PMC6030493

[90] Liu Y Gu W . p53 in ferroptosis regulation: The new weapon for the old guardian. Cell Death Differ. (2022) 29:895–910. doi: 10.1038/s41418-022-00943-y 35087226 PMC9091200

[91] Xie Y Zhu S Song X Sun X Fan Y Liu J . The tumor suppressor p53 limits ferroptosis by blocking DPP4 activity. Cell Rep. (2017) 20:1692–704. doi: 10.1016/j.celrep.2017.07.055 28813679

[92] Chen F Kang R Liu J Tang D . The ACSL4 network regulates cell death and autophagy in diseases. Biol (Basel). (2023) 12:864. doi: 10.3390/biology12060864 37372148 PMC10295397

[93] Liu X Hai Y Dong J Xu L Hou W Su J . Realgar-induced KRAS mutation lung cancer cell death via KRAS/Raf/MAPK mediates ferroptosis. Int J Oncol. (2022) 61:157. doi: 10.3892/ijo.2022.5447 36321791 PMC9635866

[94] Li YJ Fahrmann JF Aftabizadeh M Zhao Q Tripathi SC Zhang C . Fatty acid oxidation protects cancer cells from apoptosis by increasing mitochondrial membrane lipids. Cell Rep. (2022) 39:110870. doi: 10.3410/f.742175070.793595099 35649368

[95] Linkermann A Skouta R Himmerkus N Mulay SR Dewitz C De Zen F . Synchronized renal tubular cell death involves ferroptosis. Proc Natl Acad Sci USA. (2014) 111:16836–41. doi: 10.1073/pnas.1415518111 25385600 PMC4250130

[96] Pedrera L Espiritu RA Ros U Weber J Schmitt A Stroh J . Ferroptotic pores induce Ca2+ fluxes and ESCRT-III activation to modulate cell death kinetics. Cell Death Differ. (2021) 28:1644–57. doi: 10.1038/s41418-020-00691-x 33335287 PMC8167089

[97] Müller T Dewitz C Schmitz J Schröder AS Bräsen JH Stockwell BR . Necroptosis and ferroptosis are alternative cell death pathways that operate in acute kidney failure. Cell Mol Life Sci. (2017) 74:3631–45. doi: 10.1007/s00018-017-2547-4 28551825 PMC5589788

[98] Zhang Y Su SS Zhao S Yang Z Zhong CQ Chen X . RIP1 autophosphorylation is promoted by mitochondrial ROS and is essential for RIP3 recruitment into necrosome. Nat Commun. (2017) 8:14329. doi: 10.1038/ncomms14329 28176780 PMC5309790

[99] Badgley MA Kremer DM Maurer HC DelGiorno KE Lee HJ Purohit V . Cysteine depletion induces pancreatic tumor ferroptosis in mice. Science. (2020) 368:85–9. doi: 10.1126/science.aaw9872 32241947 PMC7681911

[100] Basit F van Oppen LM Schöckel L Bossenbroek HM van Emst-de Vries SE Hermeling JC . Mitochondrial complex I inhibition triggers a mitophagy-dependent ROS increase leading to necroptosis and ferroptosis in melanoma cells. Cell Death Dis. (2017) 8:e2716. doi: 10.1038/cddis.2017.133 28358377 PMC5386536

[101] Wang Z Guo LM Wang Y Zhou HK Wang SC Chen D . Inhibition of HSP90α protects cultured neurons from oxygen-glucose deprivation induced necroptosis by decreasing RIP3 expression. J Cell Physiol. (2018) 233:4864–84. doi: 10.1002/jcp.26294 29334122

[102] Zhou B Liu J Kang R Klionsky DJ Kroemer G Tang D . Ferroptosis is a type of autophagy-dependent cell death. Semin Cancer Biol. (2020) 66:89–100. doi: 10.1016/j.semcancer.2019.03.002 30880243

[103] Zhou Y She R Mei Z Liu D Ge J . Crosstalk between ferroptosis and necroptosis in cerebral ischemia/reperfusion injury and Naotaifang formula exerts neuroprotective effect via HSP90-GCN2-ATF4 pathway. Phytomedicine. (2024) 130:155399. doi: 10.1016/j.phymed.2024.155399 38850632

[104] Tian Q Qin B Gu Y Zhou L Chen S Zhang S . ROS-mediated necroptosis is involved in iron overload-induced osteoblastic cell death. Oxid Med Cell Longev. (2020) 2020:1295382. doi: 10.1155/2020/1295382 33123307 PMC7586162

[105] Shi M Mobet Y Shen H . Quercetin attenuates acute kidney injury caused by cisplatin by inhibiting ferroptosis and cuproptosis. Cell Biochem Biophys. (2024) 82:2687–99. doi: 10.1007/s12013-024-01379-6 39026057

[106] Kadry MO Abdel-Megeed RM . Necroptosis and autophagy in cisplatinum-triggered nephrotoxicity: Novel insights regarding their prognostic and diagnostic potential. Toxicol Rep. (2024) 13:101807. doi: 10.1016/j.toxrep.2024.101807 39606774 PMC11600652

[107] Taha M Abdelbagi O Baokbah TAS Bagadood RM Jalal NA Obaid R . Insights into the protective effect of omega-3 nanoemulsion against colistin-induced nephrotoxicity in experimental rats: Regulation of autophagy and necroptosis via AMPK/mTOR and RIPK1/RIPK3/MLKL signaling pathways. Ren Fail. (2024) 46:2429686. doi: 10.1080/0886022x.2024.2429686 39584420 PMC11590192

[108] Lu J Wei H Yao X Chen Y Liu M Guan S . Glycidol induces necroptosis and inflammation through autophagy-necrosome pathway in renal cell and mice. Sci Total Environ. (2025) 968:178852. doi: 10.1016/j.scitotenv.2025.178852 39965374

[109] Wu W Wang X Sun Y Berleth N Deitersen J Schlütermann D . TNF-induced necroptosis initiates early autophagy events via RIPK3-dependent AMPK activation, but inhibits late autophagy. Autophagy. (2021) 17:3992–4009. doi: 10.1080/15548627.2021.1899667 33779513 PMC8726653

[110] Zhu P Hu S Jin Q Li D Tian F Toan S . Ripk3 promotes ER stress-induced necroptosis in cardiac IR injury: A mechanism involving calcium overload/XO/ROS/mPTP pathway. Redox Biol. (2018) 16:157–68. doi: 10.1016/j.redox.2018.02.019 29502045 PMC5952878

[111] She L Tu H Zhang YZ Tang LJ Li NS Ma QL . Inhibition of phosphoglycerate mutase 5 reduces necroptosis in rat hearts following ischemia/reperfusion through suppression of dynamin-related protein 1. Cardiovasc Drugs Ther. (2019) 33:13–23. doi: 10.1007/s10557-018-06848-8 30637549

[112] Xu Y Ma HB Fang YL Zhang ZR Shao J Hong M . Cisplatin-induced necroptosis in TNFα dependent and independent pathways. Cell Signal. (2017) 31:112–23. doi: 10.1016/j.cellsig.2017.01.004 28065786

[113] Wang Z Feng J Yu J Chen G . FKBP12 mediates necroptosis by initiating RIPK1-RIPK3-MLKL signal transduction in response to TNF receptor 1 ligation. J Cell Sci. (2019) 132:jcs227777. doi: 10.1242/jcs.227777 31028177

[114] Güler MC Akpinar E Tanyeli A Çomakli S Bayir Y . Costunolide prevents renal ischemia-reperfusion injury in rats by reducing autophagy, apoptosis, inflammation, and DNA damage. Iran J Basic Med Sci. (2023) 26:1168–76. doi: 10.22038/IJBMS.2023.71779.15596 37736519 PMC10510491

[115] Sun CY Nie J Zheng ZL Zhao J Wu LM Zhu Y . Renoprotective effect of scutellarin on cisplatin-induced renal injury in mice: Impact on inflammation, apoptosis, and autophagy. BioMed Pharmacother. (2019) 112:108647. doi: 10.1016/j.biopha.2019.108647 30797149

[116] Pan P Liu X Wu L Li X Wang K Wang X . TREM-1 promoted apoptosis and inhibited autophagy in LPS-treated HK-2 cells through the NF-κB pathway. Int J Med Sci. (2021) 18:8–17. doi: 10.7150/ijms.50893 33390769 PMC7738954

[117] Zheng X Chen D Wu J Gao Z Huang M Fan C . Apelin-13 inhibits ischemia-reperfusion mediated podocyte apoptosis by reducing m-TOR phosphorylation to enhance autophagy. FASEB J. (2025) 39:e70319. doi: 10.1096/fj.202402850r 39812591

[118] Gump JM Staskiewicz L Morgan MJ Bamberg A Riches DW Thorburn A . Autophagy variation within a cell population determines cell fate through selective degradation of Fap-1. Nat Cell Biol. (2014) 16:47–54. doi: 10.1038/ncb2886 24316673 PMC3876036

[119] Lindqvist LM Frank D McArthur K Dite TA Lazarou M Oakhill JS . Autophagy induced during apoptosis degrades mitochondria and inhibits type I interferon secretion. Cell Death Differ. (2018) 25:784–96. doi: 10.1038/s41418-017-0017-z 29229994 PMC5864185

[120] Rubinstein AD Eisenstein M Ber Y Bialik S Kimchi A . The autophagy protein Atg12 associates with antiapoptotic Bcl-2 family members to promote mitochondrial apoptosis. Mol Cell. (2011) 44:698–709. doi: 10.1016/j.molcel.2011.10.014 22152474

[121] Yousefi S Perozzo R Schmid I Ziemiecki A Schaffner T Scapozza L . Calpain-mediated cleavage of Atg5 switches autophagy to apoptosis. Nat Cell Biol. (2006) 8:1124–32. doi: 10.1038/ncb1482 16998475

[122] Li M Gao P Zhang J . Crosstalk between autophagy and apoptosis: Potential and emerging therapeutic targets for cardiac diseases. Int J Mol Sci. (2016) 17:332. doi: 10.3390/ijms17030332 26950124 PMC4813194

[123] Li X Su J Xia M Li H Xu Y Ma C . Caspase-mediated cleavage of Beclin1 inhibits autophagy and promotes apoptosis induced by S1 in human ovarian cancer SKOV3 cells. Apoptosis. (2016) 21:225–38. doi: 10.1007/s10495-015-1197-y 26573276

[124] Chien CT Shyue SK Lai MK . Bcl-xL augmentation potentially reduces ischemia/reperfusion induced proximal and distal tubular apoptosis and autophagy. Transplantation. (2007) 84:1183–90. doi: 10.1097/01.tp.0000287334.38933.e3 17998875

[125] Yang Z Zhong Z Li M Xiong Y Wang Y Peng G . Hypothermic machine perfusion increases A20 expression which protects renal cells against ischemia/reperfusion injury by suppressing inflammation, apoptosis and necroptosis. Int J Mol Med. (2016) 38:161–71. doi: 10.3892/ijmm.2016.2586 27177159 PMC4899006

[126] Eleftheriadis T Pissas G Antoniadi G Liakopoulos V Stefanidis I . Cell death patterns due to warm ischemia or reperfusion in renal tubular epithelial cells originating from human, mouse, or the native hibernator hamster. Biol (Basel). (2018) 7:48. doi: 10.3390/biology7040048 30445750 PMC6316155

[127] Martin-Sanchez D Fontecha-Barriuso M Carrasco S Sanchez-Niño MD Mässenhausen AV Linkermann A . TWEAK and RIPK1 mediate a second wave of cell death during AKI. Proc Natl Acad Sci USA. (2018) 115:4182–7. doi: 10.1073/pnas.1716578115 29588419 PMC5910825

[128] Kruidering M Evan GI . Caspase-8 in apoptosis: The beginning of "the end"? IUBMB Life. (2000) 50:85–90. doi: 10.1080/713803693 11185963

[129] Vanden Berghe T Kaiser WJ Bertrand MJ Vandenabeele P . Molecular crosstalk between apoptosis, necroptosis, and survival signaling. Mol Cell Oncol. (2015) 2:e975093. doi: 10.4161/23723556.2014.975093 27308513 PMC4905361

[130] Holler N Zaru R Micheau O Thome M Attinger A Valitutti S . Fas triggers an alternative, caspase-8-independent cell death pathway using the kinase RIP as effector molecule. Nat Immunol. (2000) 1:489–95. doi: 10.1038/82732 11101870

[131] Zhang J Chan FK . Cell biology. RIPK3 takes another deadly turn. Science. (2014) 343:1322–3. doi: 10.1126/science.1252526 24653026 PMC4282744

[132] Kusunoki Y Li C Long H Watanabe-Kusunoki K Kuang M Marschner JA . Gasdermin D deficiency aggravates nephrocalcinosis-related chronic kidney disease with rendering macrophages vulnerable to necroptosis. Cell Death Dis. (2025) 16:283. doi: 10.1038/s41419-025-07620-1 40221396 PMC11993636

[133] Zhang R Guan S Meng Z Zhang D Lu J . Ginsenoside Rb1 alleviates 3-MCPD-induced renal cell pyroptosis by activating mitophagy. Food Chem Toxicol. (2024) 186:114522. doi: 10.1016/j.fct.2024.114522 38373586

[134] Jiayi H Ziyuan T Tianhua X Mingyu Z Yutong M Jingyu W . Copper homeostasis in chronic kidney disease and its crosstalk with ferroptosis. Pharmacol Res. (2024) 202:107139. doi: 10.1016/j.phrs.2024.107139 38484857

[135] Zheng CM Hou YC Tsai KW Hu WC Yang HC Liao MT . Resveratrol mitigates uremic toxin-induced intestinal barrier dysfunction in chronic kidney disease by promoting mitophagy and inhibiting apoptosis pathways. Int J Med Sci. (2024) 21:2437–49. doi: 10.7150/ijms.100963 39439463 PMC11492888

[136] Zhu Y Cui H Xia Y Gan H . RIPK3-mediated necroptosis and apoptosis contributes to renal tubular cell progressive loss and chronic kidney disease progression in rats. PloS One. (2016) 11:e0156729. doi: 10.1371/journal.pone.0156729 27281190 PMC4900656

[137] Guo Y Zhao Y Qiao Y Xing Y Fang Y Zhao Y . Targeting panoptosis: A narrative review of its therapeutic potential in kidney disease. BMC Nephrol. (2025) 26:545. doi: 10.1186/s12882-025-04339-1 41023669 PMC12481750

[138] Xie X Lou H Shi Y Gan G Deng H Ma X . A network pharmacological-based study of the mechanism of Liuwei Dihuang pill in the treatment of chronic kidney disease. Med (Baltimore). (2023) 102:e33727. doi: 10.1097/MD.0000000000033727 37171332 PMC10174353

[139] Wang X Li Q Sui B Xu M Pu Z Qiu T . Schisandrin A from Schisandra chinensis attenuates ferroptosis and NLRP3 inflammasome-mediated pyroptosis in diabetic nephropathy through mitochondrial damage by AdipoR1 ubiquitination. Oxid Med Cell Longev. (2022) 2022:5411462. doi: 10.1155/2022/5411462 35996380 PMC9391610

[140] Wang X Wang W Han M Zhang J Li Y . ELAVL1-stabilized USP22 promotes diabetic nephropathy progression via mediating podocyte injury and death by triggering ACSL4 deubiquitination. Transpl Immunol. (2025) 93:102280. doi: 10.1016/j.trim.2025.102280 40930301

[141] Liang H Chen S Zhong J Chen X Xie T Chen Z . Dapagliflozin improves diabetic nephropathy by regulating autophagy and ferroptosis through CYP450s/ROS pathway. Biochem Pharmacol. (2025) 240:117134. doi: 10.1016/j.bcp.2025.117134 40639443

[142] Ji H Zhao Y Ma X Wu L Guo F Huang F . Upregulation of UHRF1 promotes PINK1-mediated mitophagy to alleviates ferroptosis in diabetic nephropathy. Inflammation. (2024) 47:718–32. doi: 10.1007/s10753-023-01940-0 38055118

[143] Zheng GS Tan YM Shang YY Liu YP Hu BA Wang D . CIDEC silencing attenuates diabetic nephropathy via inhibiting apoptosis and promoting autophagy. J Diabetes Investig. (2021) 12:1336–45. doi: 10.1111/jdi.13534 33655702 PMC8354488

[144] Liu J Chang A Peng H Huang H Hu P Yao A . Isoferulic acid regulates CXCL12/CXCR4-mediated apoptosis and autophagy in podocyte and mice with STZ-induced diabetic nephropathy. Int Immunopharmacol. (2025) 144:113707. doi: 10.1016/j.intimp.2024.113707 39616856

[145] Lv Z Hu J Su H Yu Q Lang Y Yang M . TRAIL induces podocyte PANoptosis via death receptor 5 in diabetic kidney disease. Kidney Int. (2025) 107:317–31. doi: 10.1016/j.kint.2024.10.026 39571905

[146] Toffoli B Tonon F Tisato V Michelli A Zauli G Secchiero P . TRAIL treatment prevents renal morphological changes and TGF-β-induced mesenchymal transition associated with diabetic nephropathy. Clin Sci (Lond). (2020) 134:2337–52. doi: 10.1042/CS20201004 32857135

[147] Wen S Deng F Li L Xu L Li X Fan Q . VX-765 ameliorates renal injury and fibrosis in diabetes by regulating caspase-1-mediated pyroptosis and inflammation. J Diabetes Investig. (2022) 13:22–33. doi: 10.1111/jdi.13660 34494385 PMC8756311

[148] Xia W Li Y Wu M Jin Q Wang Q Li S . Gasdermin E deficiency attenuates acute kidney injury by inhibiting pyroptosis and inflammation. Cell Death Dis. (2021) 12:139. doi: 10.1038/s41419-021-03431-2 33542198 PMC7862699

[149] Su Y Wang Y Liu M Chen H . Hydrogen sulfide attenuates renal I/R-induced activation of the inflammatory response and apoptosis via regulating Nrf2-mediated NLRP3 signaling pathway inhibition. Mol Med Rep. (2021) 24:518. doi: 10.3892/mmr.2021.12157 34013370 PMC8160482

[150] Pan LL Liang W Ren Z Li C Chen Y Niu W . Cathelicidin-related antimicrobial peptide protects against ischaemia reperfusion-induced acute kidney injury in mice. Br J Pharmacol. (2020) 177:2726–42. doi: 10.1111/bph.14998 31976546 PMC7236077

[151] Cai Q Sun Z Xu S Jiao X Guo S Li Y . Disulfiram ameliorates ischemia/reperfusion-induced acute kidney injury by suppressing the caspase-11-GSDMD pathway. Ren Fail. (2022) 44:1169–81. doi: 10.1080/0886022x.2022.2098764 35837696 PMC9291718

[152] Tang Y Luo H Xiao Q Li L Zhong X Zhang J . Isoliquiritigenin attenuates septic acute kidney injury by regulating ferritinophagy-mediated ferroptosis. Ren Fail. (2021) 43:1551–60. doi: 10.1080/0886022x.2021.2003208 34791966 PMC8604484

[153] Shi Z Du Y Zheng J Tang W Liang Q Zheng Z . Liproxstatin-1 alleviated ischemia/reperfusion-induced acute kidney injury via inhibiting ferroptosis. Antioxidants (Basel). (2024) 13:182. doi: 10.3390/antiox13020182 38397780 PMC10886111

[154] Wu S Qian H Zou X Liu R . Combination of deferoxamine with cyclosporine synergistically blunt renal cold ischemia-reperfusion injury in rat transplantation model. Transplant Proc. (2024) 56:1732–9. doi: 10.1016/j.transproceed.2024.08.035 39242312

[155] Huang Q Ru Y Luo Y Luo X Liu D Ma Y . Identification of a targeted ACSL4 inhibitor to treat ferroptosis-related diseases. Sci Adv. (2024) 10:eadk1200. doi: 10.1126/sciadv.adk1200 38552012 PMC10980261

[156] Tonnus W Meyer C Steinebach C Belavgeni A von Mässenhausen A Gonzalez NZ . Dysfunction of the key ferroptosis-surveilling systems hypersensitizes mice to tubular necrosis during acute kidney injury. Nat Commun. (2021) 12:4402. doi: 10.1038/s41467-021-24712-6 34285231 PMC8292346

[157] Pefanis A Bongoni AK McRae JL Salvaris EJ Fisicaro N Murphy JM . Inhibition of RIPK1 or RIPK3 kinase activity post ischemia-reperfusion reduces the development of chronic kidney injury. Biochem J. (2025) 482:73–86. doi: 10.1042/bcj20240569 39705008 PMC12220529

[158] Xu CH Wang JN Suo XG Ji ML He XY Chen X . RIPK3 inhibitor-AZD5423 alleviates acute kidney injury by inhibiting necroptosis and inflammation. Int Immunopharmacol. (2022) 112:109262. doi: 10.1016/j.intimp.2022.109262 36166972

[159] Mulay SR Desai J Kumar SV Eberhard JN Thomasova D Romoli S . Cytotoxicity of crystals involves RIPK3-MLKL-mediated necroptosis. Nat Commun. (2016) 7:10274. doi: 10.1038/ncomms10274 26817517 PMC4738349

[160] Liu XQ Liu MM Jiang L Gao L Zhang Y Huang YB . A novel small molecule Hsp90 inhibitor, C-316-1, attenuates acute kidney injury by suppressing RIPK1-mediated inflammation and necroptosis. Int Immunopharmacol. (2022) 108:108849. doi: 10.1016/j.intimp.2022.108849 35588657

[161] Zhang DD . Mechanistic studies of the Nrf2-Keap1 signaling pathway. Drug Metab Rev. (2006) 38:769–89. doi: 10.1080/03602530600971974 17145701

[162] Long C Yang J Yang H Li X Wang G . Attenuation of renal ischemia/reperfusion injury by oleanolic acid preconditioning via its antioxidant, anti-inflammatory, and anti-apoptotic activities. Mol Med Rep. (2016) 13:4697–704. doi: 10.3892/mmr.2016.5128 27082705

[163] Zheng Q Xing J Li X Tang X Zhang D . PRDM16 suppresses ferroptosis to protect against sepsis-associated acute kidney injury by targeting the NRF2/GPX4 axis. Redox Biol. (2024) 78:103417. doi: 10.1016/j.redox.2024.103417 39549609 PMC11612791

[164] Pang Y Zhang PC Lu RR Li HL Li JC Fu HX . Andrade-Oliveira salvianolic acid B modulates caspase-1-mediated pyroptosis in renal ischemia-reperfusion injury via Nrf2 pathway. Front Pharmacol. (2020) 11:541426. doi: 10.3389/fphar.2020.541426 33013384 PMC7495093

[165] Diao C Chen Z Qiu T Liu H Yang Y Liu X . Inhibition of PRMT5 attenuates oxidative stress-induced pyroptosis via activation of the Nrf2/HO-1 signal pathway in a mouse model of renal ischemia-reperfusion injury. Oxid Med Cell Longev. (2019) 2019:2345658. doi: 10.1155/2019/2345658 31885778 PMC6899313

[166] Zhang B Wan S Liu H Qiu Q Chen H Chen Z . Naringenin alleviates renal ischemia reperfusion injury by suppressing ER stress-induced pyroptosis and apoptosis through activating Nrf2/HO-1 signaling pathway. Oxid Med Cell Longev. (2022) 2022:5992436. doi: 10.1155/2022/5992436 36262286 PMC9576412

[167] Wu QQ Wang Y Senitko M Meyer C Wigley WC Ferguson DA . Bardoxolone methyl (BARD) ameliorates ischemic AKI and increases expression of protective genes Nrf2, PPARγ, and HO-1. Am J Physiol Renal Physiol. (2011) 300:F1180–92. doi: 10.1152/ajprenal.00353.2010 21289052 PMC3094059

[168] Zaghlool SS Abdelaal N El-Shoura EAM Mahmoud NI Ahmed YM . Restoring glomerular filtration rate by sulforaphane modulates ERK1/2/JNK/p38MAPK, IRF3/iNOS, Nrf2/HO-1 signaling pathways against folic acid-induced acute renal injury in rats. Int Immunopharmacol. (2023) 123:110777. doi: 10.1016/j.intimp.2023.110777 37567014

[169] Ji Y Du S Li J Ma H Wang X Hao Y . Discovery of Zharp1-163 as a dual inhibitor of ferroptosis and necroptosis for the treatment of inflammatory disorders and kidney injury. Cell Death Discov. (2025) 11:413. doi: 10.1038/s41420-025-02693-5 40877228 PMC12394658

[170] Radajewska A Szyller J Krzywonos-Zawadzka A Olejnik A Sawicki G Bil-Lula I . Mitoquinone alleviates donation after cardiac death kidney injury during hypothermic machine perfusion in rat model. Int J Mol Sci. (2023) 24:14772. doi: 10.3390/ijms241914772 37834219 PMC10572969

[171] Liu X Murphy MP Xing W Wu H Zhang R Sun H . Mitochondria-targeted antioxidant MitoQ reduced renal damage caused by ischemia-reperfusion injury in rodent kidneys: Longitudinal observations of T2 -weighted imaging and dynamic contrast-enhanced MRI. Magn Reson Med. (2018) 79:1559–67. doi: 10.1002/mrm.26772 28608403 PMC5811825

[172] Liu Y Xu D Wang L Du W Zhang L Xiang X . MBTPS2 exacerbates albuminuria in streptozotocin-induced type I diabetic nephropathy by promoting endoplasmic reticulum stress-mediated renal damage. Arch Physiol Biochem. (2022) 128:1050–7. doi: 10.1080/13813455.2020.1749084 32255378

[173] Wang J Sun Y Liu J Yang B Wang T Zhang Z . Roles of long non-coding RNA in osteoarthritis (Review). Int J Mol Med. (2021) 48:133. doi: 10.3892/ijmm.2021.4966 34013375 PMC8148092

[174] Liu Z Qu M Yu L Song P Chang Y . Artesunate inhibits renal ischemia-reperfusion-mediated remote lung inflammation through attenuating ROS-induced activation of NLRP3 inflammasome. Inflammation. (2018) 41:1546–56. doi: 10.1007/s10753-018-0801-z 29730819

[175] Wu W Liu D Zhao Y Zhang T Ma J Wang D . Cholecalciferol pretreatment ameliorates ischemia/reperfusion-induced acute kidney injury through inhibiting ROS production, NF-κB pathway and pyroptosis. Acta Histochem. (2022) 124:151875. doi: 10.1016/j.acthis.2022.151875 35334282

[176] Li Y Hu C Zhai P Zhang J Jiang J Suo J . Fibroblastic reticular cell-derived exosomes are a promising therapeutic approach for septic acute kidney injury. Kidney Int. (2024) 105:508–23. doi: 10.1016/j.kint.2023.12.007 38163633

[177] Tao WH Shan XS Zhang JX Liu HY Wang BY Wei X . Dexmedetomidine attenuates ferroptosis-mediated renal ischemia/reperfusion injury and inflammation by inhibiting ACSL4 via α2-AR. Front Pharmacol. (2022) 13:782466. doi: 10.3389/fphar.2022.782466 35873574 PMC9307125

[178] Sun Z Wu J Bi Q Wang W . Exosomal lncRNA TUG1 derived from human urine-derived stem cells attenuates renal ischemia/reperfusion injury by interacting with SRSF1 to regulate ASCL4-mediated ferroptosis. Stem Cell Res Ther. (2022) 13:297. doi: 10.1186/s13287-022-02986-x 35841017 PMC9284726

[179] Du YW Li XK Wang TT Zhou L Li HR Feng L . Cyanidin-3-glucoside inhibits ferroptosis in renal tubular cells after ischemia/reperfusion injury via the AMPK pathway. Mol Med. (2023) 29:42. doi: 10.1186/s10020-023-00642-5 37013504 PMC10069074

[180] Yang J Sun X Huang N Li P He J Jiang L . Entacapone alleviates acute kidney injury by inhibiting ferroptosis. FASEB J. (2022) 36:e22399. doi: 10.1096/fj.202200241rr 35691001

[181] von Mässenhausen A Tonnus W Himmerkus N Parmentier S Saleh D Rodriguez D . Phenytoin inhibits necroptosis. Cell Death Dis. (2018) 9:359. doi: 10.1038/s41419-018-0394-3 29500402 PMC5834524

[182] Chen Z Li Y Yuan Y Lai K Ye K Lin Y . Single-cell sequencing reveals homogeneity and heterogeneity of the cytopathological mechanisms in different etiology-induced AKI. Cell Death Dis. (2023) 14:318. doi: 10.1038/s41419-023-05830-z 37169762 PMC10175265

[183] Schiffer TA Gustafsson H Palm F . Kidney outer medulla mitochondria are more efficient compared with cortex mitochondria as a strategy to sustain ATP production in a suboptimal environment. Am J Physiol Renal Physiol. (2018) 315:F677–81. doi: 10.1152/ajprenal.00207.2018 29846107

[184] Sanz AB Sanchez-Niño MD Ramos AM Ortiz A . Regulated cell death pathways in kidney disease. Nat Rev Nephrol. (2023) 19:281–99. doi: 10.1038/s41581-023-00694-0 36959481 PMC10035496

[185] Ostermann M Zarbock A Goldstein S Kashani K Macedo E Murugan R . Recommendations on acute kidney injury biomarkers from the Acute Disease Quality Initiative Consensus Conference: A consensus statement. JAMA Netw Open. (2020) 3:e2019209. doi: 10.1001/jamanetworkopen.2020.19209 33021646

[186] Liu D Du Y Jin FY Xu XL Du YZ . Renal cell-targeted drug delivery strategy for acute kidney injury and chronic kidney disease: A mini-review. Mol Pharm. (2021) 18:3206–22. doi: 10.1021/acs.molpharmaceut.1c00511 34337953

[187] Lake BB Chen S Hoshi M Plongthongkum N Salamon D Knoten A . A single-nucleus RNA-sequencing pipeline to decipher the molecular anatomy and pathophysiology of human kidneys. Nat Commun. (2019) 10:2832. doi: 10.1038/s41467-019-10861-2 31249312 PMC6597610

[188] Phuycharoen M Zarrineh P Bridoux L Amin S Losa M Chen K . Uncovering tissue-specific binding features from differential deep learning. Nucleic Acids Res. (2020) 48:e27. doi: 10.1093/nar/gkaa009 31974574 PMC7049686

[189] Zhou Z Zhang D Wang Y Liu C Wang L Yuan Y . Urinary exosomes: a promising biomarker of drug-induced nephrotoxicity. Front Med (Lausanne). (2023) 10:1251839. doi: 10.3389/fmed.2023.1251839 37809338 PMC10556478

[190] Albert C Haase M Albert A Zapf A Braun-Dullaeus RC Haase-Fielitz A . Biomarker-guided risk assessment for acute kidney injury: Time for clinical implementation? Ann Lab Med. (2021) 41:1–15. doi: 10.3343/alm.2021.41.1.1 32829575 PMC7443517

[191] Li H Ouyang Y Lv H Liang H Luo S Zhang Y . Nanoparticle-mediated Klotho gene therapy prevents acute kidney injury to chronic kidney disease transition through regulating PPARα signaling in renal tubular epithelial cells. Biomaterials. (2025) 315:122926. doi: 10.1016/j.biomaterials.2024.122926 39500111

[192] Liang X Liu H Hu H Ha E Zhou J Abedini A . TET2 germline variants promote kidney disease by impairing DNA repair and activating cytosolic nucleotide sensors. Nat Commun. (2024) 15:9621. doi: 10.1038/s41467-024-53798-x 39511169 PMC11543665

